# LncRNA AP000654.1 attenuates cerebral ischemia-reperfusion injury by regulating mitophagy-mediated NLRP3 inflammasome activation

**DOI:** 10.1186/s12967-026-08541-z

**Published:** 2026-07-07

**Authors:** Chi Zhang, Yixiang Cui, Zhaojing Zhang, Manyue Zhang, Qiong Zhang, Yanyang Huang, Yatian Xu, Tianyi Han, Dongzhi Yang, Ying He

**Affiliations:** 1 Department of Medical Genetics and Cell Biology, School of Basic Medical Sciences, Zhengzhou University, Zhengzhou, China; 2 School of Life Sciences, Zhengzhou University, Zhengzhou, 450001 China

**Keywords:** lncRNA AP000654.1, Ischemic stroke, Mitophagy, NLRP3 inflammasome, Microglia, Neuroinflammation

## Abstract

**Background:**

Ischemic stroke (IS) remains a leading cause of disability worldwide, and its therapeutic window is very narrow. Ischemia-reperfusion injury exacerbates secondary brain damage. Microglial NLRP3 inflammasome activation drives neuroinflammation, yet its upstream regulation requires further elucidation. We previously identified lncRNA AP000654.1 as significantly downregulated in the peripheral blood of stroke patients, prompting our investigation into its potential role in microglial biology.

**Methods:**

Using an in vitro oxygen-glucose deprivation/reperfusion (OGD/R) model in HMC3 human microglial cells, we investigated the role of lncRNA AP000654.1—previously identified as significantly downregulated in peripheral blood of stroke patients—in regulating microglial inflammatory responses.

**Results:**

OGD/R treatment upregulated NLRP3 inflammasome-related genes, induced pyroptosis-associated changes, and altered mitophagy markers. LncRNA AP000654 0.1 overexpression suppressed this excessive mitophagy by inhibiting the PINK1-Parkin pathway, thereby improving mitochondrial function. This suppression subsequently attenuated NLRP3 inflammasome assembly and pyroptosis. Crucially, enforcing mitophagy with rapamycin exacerbated the injury, which was effectively rescued by lncRNA AP000654.

**Conclusion:**

LncRNA AP000654.1 is associated with suppression of excessive mitophagy-assocated mitochondrial stress and NLRP3 inflammasome activation in microglia, suggesting its potential relevance as a therapeutic target in ischemic stroke.

**Graphical Abstract:**

**Figure:**
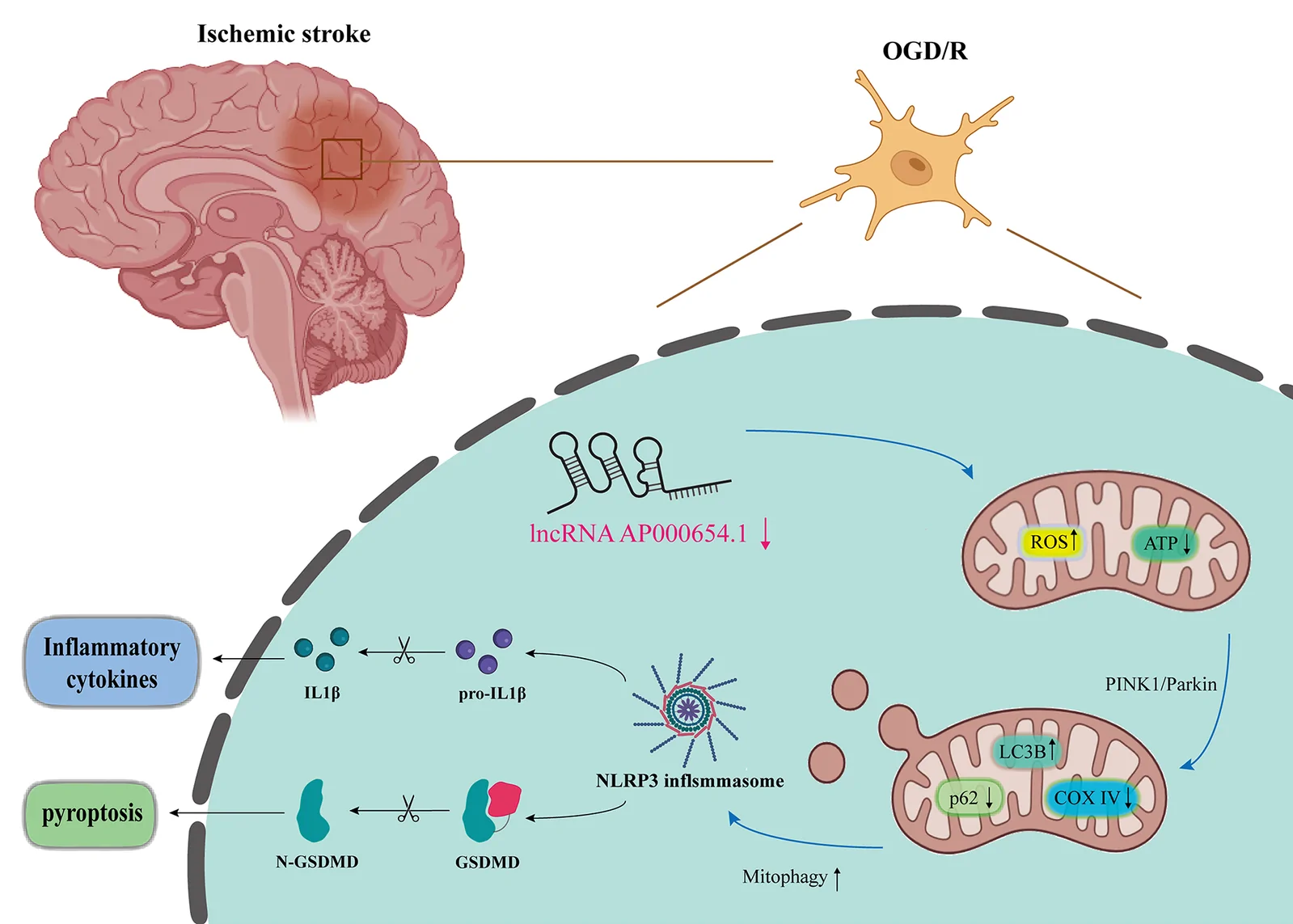

**Supplementary Information:**

The online version contains supplementary material available at https://doi.org/10.1186/s12967-026-08541-z.

## Introduction

Ischemic stroke (IS), resulting from cerebral blood flow obstruction, accounts for approximately 87% of all stroke cases and remains the primary focus of stroke research [1]. It involves highly complex pathophysiological mechanisms, among which neuroinflammation has emerged as a key driver of secondary brain injury that markedly exacerbates neuronal loss and dysfunction [2]. At present, reperfusion-based strategies—including intravenous thrombolysis with tissue plasminogen activator (tPA) and mechanical thrombectomy—represent the only approved treatments for acute ischemic stroke (IS). However, due to narrow time windows for treatment, fewer than 10% of patients qualify for these therapies. Moreover, even when blood flow is successfully restored, it does not guarantee that neurological function will not worsen later. Indeed, restoration of blood flow paradoxically triggers secondary neuroinflammatory damage. This limitation leaves a major unmet clinical need, requiring new therapeutic strategies that specifically target and suppress post-ischemic neuroinflammation [3, 4].

Following ischemic insult, an irreversible infarct core forms, surrounded by a penumbral region that is vulnerable yet potentially salvageable. Reperfusion triggers the release of damage-associated molecular patterns (DAMPs) from injured neurons and glial cells, which activate pattern recognition receptors (PRRs) and initiate inflammatory cascades. This response rapidly upregulates proinflammatory cytokines (e.g., IL-1β, IL-18, IL-6, TNF-α), thereby amplifying neuronal injury and enlarging infarct volume [5, 6]. These processes are particularly prominent in microglia, the resident immune cells of the brain, which serve as primary mediators of neuroinflammatory responses following ischemic injury [7, 8]. Experimental studies have demonstrated that suppressing microglial overactivation or modulating inflammasome activity mitigates neuroinflammation, reduces infarct volume, and enhances functional recovery post-ischemic stroke [9, 10]. Thus, targeting microglia and associated inflammatory pathways has emerged as a promising therapeutic strategy to limit secondary brain injury.

Central to microglial neuroinflammation is the NLRP3 inflammasome. Upon activation, NLRP3 assembles with ASC and pro-caspase-1, leading to caspase-1 activation and the processing of pro-IL-1β and pro-IL-18 into their mature biologically active forms [11]. In addition to releasing cytokines, this cascade leads to pyroptosis—a form of programmed cell death that is powerful, pro-inflammatory, and causes the cell to break open [12]. Targeting microglial activation and NLRP3 inflammasome signaling has thus garnered significant attention as a therapeutic approach for IS [13–15].

Mitochondrial dysfunction is a primary activator of the NLRP3 inflammasome [16]. During ischemia-reperfusion, damaged mitochondria release mitochondrial reactive oxygen species (mtROS) and mitochondrial DNA (mtDNA), which serve as potent triggers for NLRP3 assembly. Ding et al. found that aldosterone stimulates mtROS production, which mediates NLRP3 activation and induces renal tubular cell damage [17]. Nakahira et al. showed that LPS + ATP treatment of macrophages activates caspase-1, induces cytoplasmic translocation of mtDNA (a process requiring mtROS), and that cytoplasmic mtDNA enhances the expression of downstream inflammatory factors [18]. Juliana et al. noted that LPS + ATP activates NLRP3 in macrophages, whereas mtROS scavenging inhibits this activation, indicating mtROS’s role [19]. Given these observations, timely clearance of dysfunctional mitochondria is therefore crucial for alleviating excessive inflammatory responses. Notably, although mitochondria‑mediated NLRP3 activation has been well characterized in peripheral cell types and non‑neuronal systems, accumulating evidence indicates that analogous mechanisms function in microglia under ischemic stress, thereby contributing to the progression of post‑stroke neuroinflammation.

Mitophagy, a selective form of autophagy responsible for eliminating damaged mitochondria, is essential for maintaining mitochondrial homeostasis and limiting the release of mitochondria-derived pro-inflammatory signals [20]. Among the currently recognized mitophagy pathways, the PINK1–Parkin pathway is the best characterized. Under conditions of mitochondrial depolarization, PINK1 accumulates on the outer mitochondrial membrane and recruits Parkin, which ubiquitinates mitochondrial proteins and promotes their recognition by autophagy adaptors such as p62 and NDP52, thereby facilitating mitochondrial clearance [21, 22]. Although basal mitophagy generally exerts protective effects, the consequences of mitophagy activation following ischemia–reperfusion appear to be context dependent. Both insufficient mitochondrial quality control and excessive mitochondrial turnover have been associated with pathological injury [23–26]. These observations suggest that the biological effects of mitophagy depend not only on whether it is activated, but also on the extent and timing of its activation. Therefore, identifying endogenous regulators that modulate mitophagy within an appropriate range may be important for limiting secondary injury after ischemic stroke.

Long non-coding RNAs (lncRNAs) have emerged as important regulators of cellular stress responses, including those involved in cerebral ischemia [27, 28]. In our previous clinical screening, we observed that LncRNA AP000654.1 was significantly downregulated in the peripheral blood of patients with IS, implying a potential role in disease pathogenesis. In addition, our preliminary data indicated that AP000654.1 is predominantly expressed in microglia. However, its specific function and molecular mechanism in ischemic stroke remain unclear. Given that PINK1–Parkin pathway associated mitophagy is a central pathway for mitochondrial quality control and has been reported to influence NLRP3 inflammasome activation through mitochondrial reactive oxygen species (mtROS) and mitochondrial DNA (mtDNA)-related signaling [29, 30]. Accordingly, we hypothesized that lncRNA AP000654.1 may exert neuroprotective effects, at least in part, through regulation of mitophagy-associated mitochondrial stress responses, thereby suppressing NLRP3 inflammasome activation in microglia.

To validate this hypothesis, we employed HMC3 cells (Human Micro glial Clone 3), a human microglial cell line, to establish an oxygen-glucose deprivation/reoxygenation (OGD/R) model. Results demonstrated that overexpression of the long non-coding RNA AP000654.1 mitigated OGD/R-induced damage. Mechanistically, we discovered that this long non-coding RNA exerts its effects by inhibiting PINK1-Parkin-mediated excessive mitochondrial autophagy, thereby blocking the mtROS/NLRP3/GSDMD signaling axis. Crucially, the mitochondrial autophagy activator rapamycin (RAPA) not only exacerbated injury but also partially reversed the protective effects of LncRNA AP000654.1, providing direct causal evidence for its role in this pathway. This study reveals long non-coding RNA AP000654.1 as a novel endogenous regulator of the mitochondrial autophagy-NLRP3 axis, offering a fresh perspective and potential therapeutic target for ischemic stroke treatment.

## Materials and methods

### Reagents and instruments

Human microglial cells (HMC3, Human Micro glial Clone 3; ATCC, CRL-3304) were obtained from Prof. Qiao Hailing’s group (Zhengzhou University, China). cells are an immortalized human microglial cell line derived from primary human fetal brain tissue. Reagents comprised TRNzol Universal reagent (TIANGEN, Germany, Catalog Number: DP424), antibodies (Abmart, Proteintech, HUABIO, ABclonal), and detection kits for ATP/ROS/Hoechst 33,342/PI (Beyotime, China). Lentiviral plasmids (plvx-IRES-puro) carrying LncRNA AP000654.1 or negative control (NC) were designed by Tsingke Biotechnology. Antibodies used were P62/SQSTM1 Antibody (Abmart|T55546F|1:1000|WB), NLRP3 Antibody (Abmart|T55651|1:1000|WB), Total and cleaved N-terminal GSDMD Antibody(Abmart|P30823|1:1000|WB), LC3B Recombinant Rabbit Monoclonal Antibody (HUABIO|ET1701-65|1:3000|WB, |1:2000|IHC), COX IV Recombinant Rabbit Monoclonal Antibody (HUABIO|ET1701-63|1:2000|WB, |1:500|IHC), Caspase-1 p20 Rabbit pAb (Abmart|PC6368|1:1000|WB), PINK1 Rabbit Antibody (Abmart|PK05715|1:1000|WB), Parkin Rabbit Antibody (Abmart|T56641|1:1000|WB), Beclin 1 Recombinant Rabbit Monoclonal Antibody (HUABIO|HA721216|1:5000|WB), β-actin Rabbit Antibody (ABclonal|AC038|1:1000|WB), 488-Goat Anti-Rabbit Antibody (Proteintech|RGAR002|1:500|IF), 594-Goat Anti-Mouse Antibody (Proteintech|RGAM004|1:500|IF).

### Cell culture and treatment

HMC3 cells were maintained in MEM (PM150410, Procell) supplemented with 10% fetal bovine serum (FBS, Biological Industry) and 1% penicillin/streptomycin (SC118-01, Seven, Beijing, China) (37 °C, 5% CO2). The oxygen-glucose deprivation/reperfusion (OGD/R) conditions (2 h hypoxia followed by 4 h reperfusion) were selected based on previously established protocols demonstrating robust induction of inflammatory responses in microglial cells without complete cell death [31]. cells in glucose-free DMEM (BC-M-038, Biochannel) were incubated under hypoxia (10% H₂, 5% CO₂, 85% N₂) for 2 h, followed by reperfusion with complete medium for 4 h.

### Lentivirus transduction

Lentivirus packaging: 293T cells were transfected with plasmids (plvx-IRES-puro-AP000654.1/OE, psPAX, pMD2.G) using PEI. After 48 h, supernatants containing lentivirus were collected, filtered (0.45 μm), and stored at − 80 °C. HMC3 cells were transduced with OE or NC lentivirus in medium containing 5 µg/mL polybrene (H8761, Solarbio, Beijing, China)0.2 µg/mL Puromycin Dihydrochloride (IP12803, Solarbio, Beijing, China) selection confirmed stable infection.

### RNA extraction and quantitative PCR (q-PCR)

Total RNA was extracted using TRNzol Universal reagent (Tiangen Biotech, Catalog Number: DP424), and the cDNA was generated with HiScript II Reverse Transcriptase (Vazyme, Catalog Number: R222-01) according to the instructions. q-PCR was performed with Taq Pro Universal SYBR qPCR Master Mix (Vazyme, Catalog Number: Q712) using QuantStudio™ 5 (ThermoFisher). The data were analyzed by the 2 − ΔΔCt method, and relative mRNA expression was normalized to β-actin. The primer sequences used were as follows: for cells, LncRNA AP000654.1-fwd (5′-CCAGATGCCAGTTATGTTCTCTG­3′) and LncRNA AP000654.1-rev (5’-CTAAGTCCGCCTAAGCTAGGG-3’); β-actin­fwd (5’-GGAAATCGTGCGTGACATTAA-3’) and β-actin­rev (5’- AGGAAGGAAGGCTGGAAGAG-3’); NLRP3-fwd (5’-GATCTTCGCTGCGATCAACAG-3’) and NLRP3-rev (5’-CGTGCATTATCTGAACCCCAC-3’); IL1β­fwd (5’-ATGATGGCTTATTACAGTGGCAA-3’) and IL1β­rev (5’-GTCGGAGATTCGTAGCTGGA-3’); GSDMD-fwd (5’-GTGTGTCAACCTGTCTATCAAGG-3’) and GSDMD-rev (5’-CATGGCATCGTAGAAGTGGAAG-3’).

### Western blotting

The cells were harvested and lysed in RIPA buffer supplemented with protease and phosphatase inhibitors (Solarbio, Beijing, China, Catalog Number: R0010). Total proteins were resolved by sodium dodecyl sulfate-polyacrylamide gel electrophoresis (SDS-PAGE) using a rapid gel preparation kit (Seven, Beijing, China). Proteins were subsequently transferred onto polyvinylidene difluoride (PVDF) membranes (Millipore, Immobilon IPVH00010 & ISEQ00010). Membranes were blocked with 5% non-fat milk in Tris-buffered saline containing 0.1% Tween-20 (TBST) for 1 h at room temperature, followed by overnight incubation at 4 °C with primary antibodies diluted in blocking buffer according to manufacturers’ specifications. After three washes with TBST, membranes were probed with horseradish peroxidase (HRP)-conjugated secondary antibodies (goat anti-rabbit or goat anti-mouse IgG) for 2 h at room temperature. Immunoreactive bands were visualized using enhanced chemiluminescence (ECL) substrate (Biosharp, BL523B) and detected by a chemiluminescence imaging system (Servicebio, SCG-W3000 PLUS). Equal protein loading was verified by reprobing membranes with β-actin antibody.

### Cell immunofluorescence

Cells were seeded on lysine-coated coverslips in 24-well plates (until reaching ~ 70% confluency). After treatment, cells were washed twice with PBS (5 min per wash) and fixed with 4% paraformaldehyde (15 min). Following fixation, cells were permeabilized with 0.2% Triton X-100 in PBS (15 min) and blocked with 5% BSA in PBST (1 h). Primary antibodies were incubated overnight at 4 °C. After washing, secondary antibodies were applied for 2 h. Coverslips were mounted onto glass slides using DAPI-containing antifade medium and sealed with nail polish. Fluorescence was visualized using a confocal microscope.

### ATP assay

Cells in 6-well plates (~ 90% confluency) were lysed with 200 µL ATP lysis buffer, scraped, and centrifuged (4 °C, 12,000 ×g, 5 min). The supernatant was collected for analysis. ATP standards (0.625–10 µM) were prepared by serial dilution. ATP detection working solution was mixed with samples or standards, and luminescence was measured using a microplate reader. ATP levels were normalized to total protein concentration (determined via BCA assay) and expressed as nmol/mg protein.

### ROS detection

Cells were cultured in 24-well plates for 48 h (~ 80% confluency). After removing the medium, cells were washed twice with PBS and incubated with 10 µM DCFH-DA (diluted 1:1000 in serum-free medium) at 37 °C for 20 min. Post-incubation, cells were washed three times with serum-free medium. Fluorescence images were captured using an Olympus FV-3000 microscope, and mean intensity was quantified via ImageJ.

### Hoechst 33,342/PI staining

Cells in 24-well plates were washed twice with PBS and stained with Hoechst 33,342/PI (1:1 ratio) in 250 µL staining buffer at 4 °C for 30 min. After washing twice with PBS, fluorescence signals were observed under a fluorescence microscope.

### RNA-FISH in mouse brain tissue

All animal experiments were conducted in accordance with the guidelines of the Institutional Animal Care and Use Committee of Zhengzhou University (Approval No. ZZUIR202605). A total of six mice were used. Six-week-old male C57BL/6 mice were injected with lncRNA AP000654.1-overexpressing or control AAV via the tail vein. After 21 days, mice were anesthetized with sodium pentobarbital (40 mg/kg) and perfused with saline followed by 4% paraformaldehyde. Brains were post-fixed in 4% paraformaldehyde (12 h), dehydrated in sucrose gradients, embedded in OCT compound, and sectioned using a cryostat. RNA-FISH was performed using probes (Boster Biological Technology) following kit instructions. Sections were hybridized with probes at 42 °C (16 h), washed with graded SSC buffers, and counterstained with DAPI. Fluorescence was analyzed via confocal microscopy.

### Autophagic flux assay

HMC3 cells were cultured on glass coverslips in 6‑well plates and transduced with Ad‑mCherry‑GFP‑LC3B adenovirus (Beyotime, C3011) at MOI 20 for 24 h. Virus‑containing medium was then replaced with fresh complete MEM, and cells were incubated for an additional 24 h to allow fusion protein expression. Following experimental treatments, coverslips were washed with PBS, fixed with 4% paraformaldehyde (15 min), and mounted with antifade medium. Images were acquired using a Leica SP8 confocal laser scanning microscope. Yellow puncta (merged GFP and mCherry signals) represent autophagosomes, whereas red‑only puncta (acid‑resistant mCherry) indicate autolysosomes.

### Statistical analysis

Data were analyzed using GraphPad Prism 9.5. Normality and homogeneity of variance were confirmed before applying Student’s t-test or one-way ANOVA. Results are expressed as mean ± SD. Statistical significance was set at *P* < 0.05.

## Results

### OGD/R treatment upregulates the transcription of NLRP3 and alters mitophagy-associated markers in HMC3 cells

**Fig. 1:**
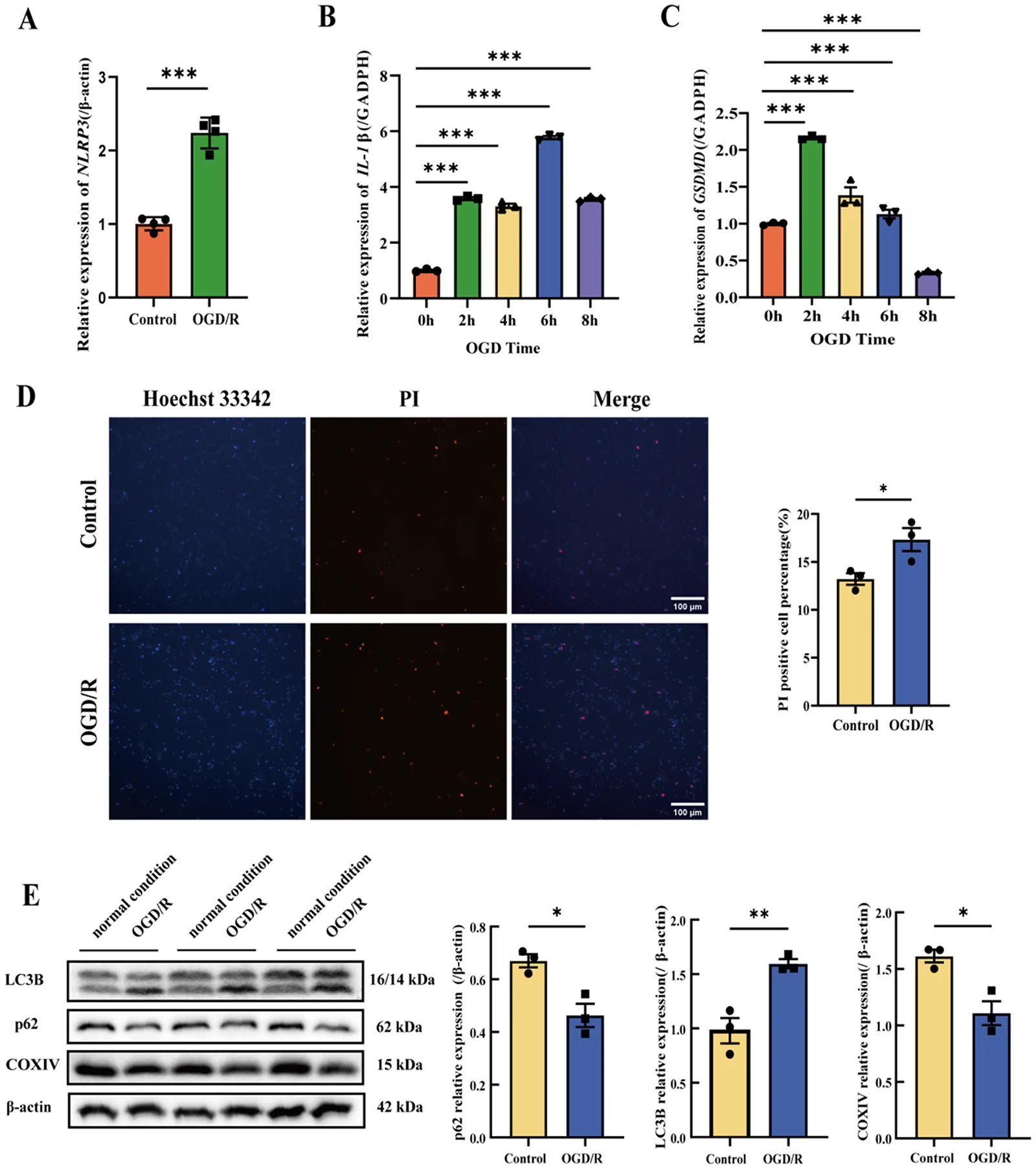
OGD/R Induces NLRP3 Inflammasome Activation, Pyroptosis-Related Gene Transcription, Membrane Permeabilization, and Mitochondrial Turnover in HMC3 Microglia. (**A**) Quantitative RT-PCR analysis of NLRP3 mRNA in HMC3 cells at the indicated time points after oxygen-glucose deprivation/reperfusion (OGD/R). NLRP3 transcription was significantly induced following OGD/R (*n* = 3). (**B** and **C**) qRT-PCR detection of the pyroptosis-associated markers IL-1β (**B**) and GSDMD (**C**) in HMC3 cells under control and OGD/R conditions (*n* = 3). (**D**) Hoechst 33,342/PI double staining to evaluate plasma membrane integrity in control and OGD/R-treated HMC3 cells. Hoechst 33,342 (blue) labels all nuclei, whereas PI (red) stains nuclei only when membrane integrity is compromised. Representative images; scale bar, 100 μm. Quantification of the number of PI-positive cells per field (10 fields per experiment, *n* = 3 independent experiments). (**E**) Western blot analysis of the autophagic marker LC3B (LC3B-II), the cargo receptor p62/SQSTM1, and the mitochondrial protein COXIV in control and OGD/R-treated HMC3 cells (*n* = 3). *P < 0.05, **P < 0.01, ***P < 0.001

To investigate the cellular response of microglia under ischemia-reperfusion (I/R) conditions, we established an in vitro oxygen-glucose deprivation/reperfusion (OGD/R) model in HMC3 human microglial cells. Quantitative PCR analysis revealed that NLRP3 mRNA expression was significantly upregulated following OGD/R treatment (Fig. 1A), with expression levels peaking at 8 h of OGD exposure (Figure S1). This transcriptional increase suggests that ischemic-like stress activates pathways associated with NLRP3-related inflammatory responses.

In parallel, the expression levels of key pyroptosis-associated molecules, IL-1β and GSDMD, were also markedly elevated under OGD/R condition (Fig. 1B and C), indicating coordinated transcriptional induction of pyroptosis-associated genes. Consistent with these molecular changes, OGD/R-treatment significantly increased the percentage of PI-positive cells compared with the control group (Fig. 1D), reflecting impaired plasma membrane integrity. While this finding is consistent with a loss of membrane barrier function, it does not by itself distinguish pyroptotic pore formation from other forms of membrane disruption. We further examined changes in autophagy- and mitochondria-related markers following OGD/R treatment. Western blot analysis showed increased LC3B levels together with reduced P62 and COXIV expression (Fig. 1E). These data indicate that OGD/R alters autophagy-related flux and mitochondrial content in HMC3 cells, which is consistent with enhanced mitochondrial turnover but does not alone establish selective mitophagy. Therefore, subsequent experiments were performed to further assess mitophagy-associated changes and their relationship to inflammatory activation.

### LncRNA AP000654.1 attenuates NLRP3 inflammasome activation in OGD/R-treated HMC3 cells

**Fig. 2:**
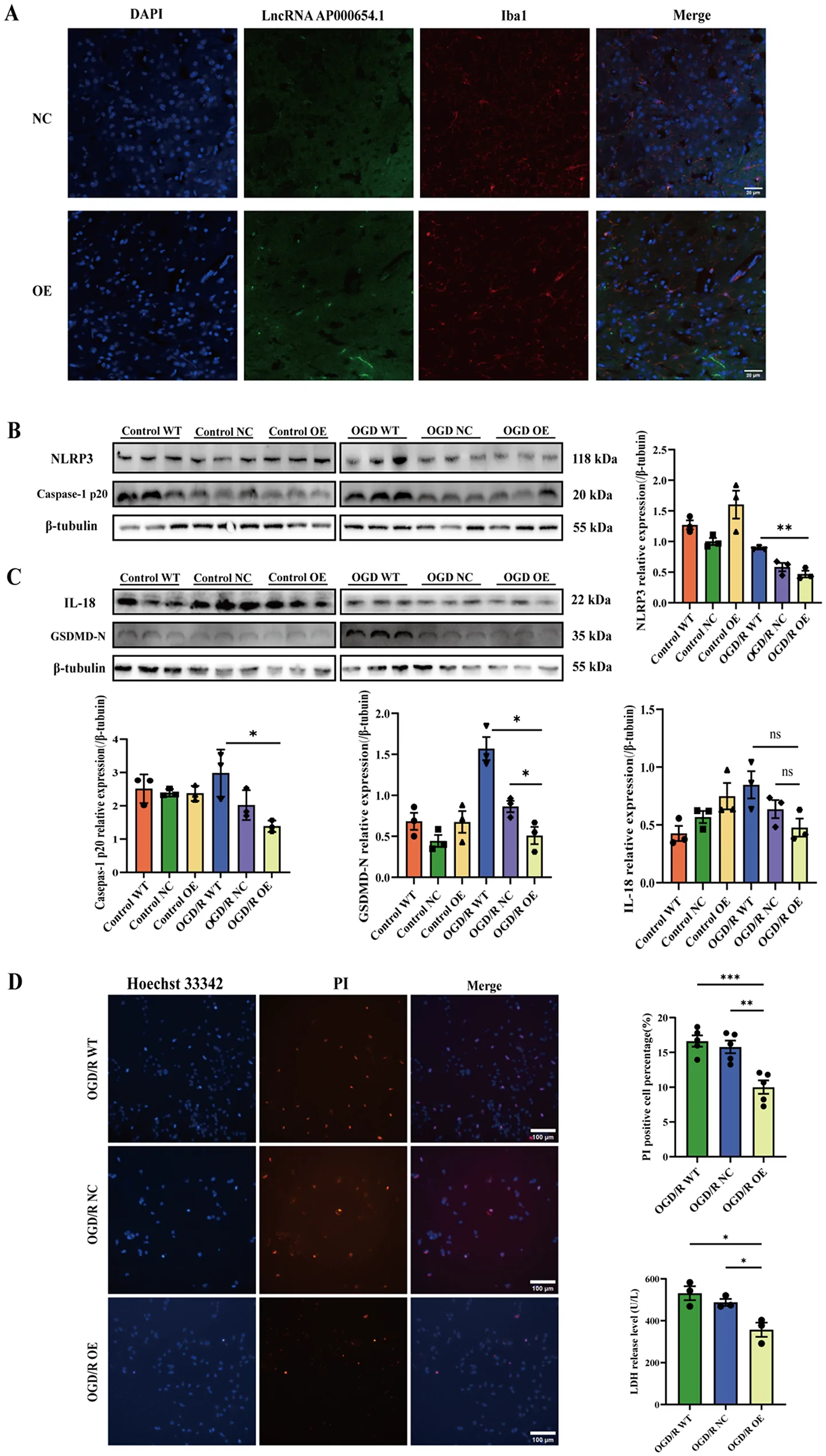
LncRNA AP000654.1 Is Expressed in Microglia and Attenuates OGD/R-Induced NLRP3 Inflammasome Activation and Pyroptosis in HMC3 Cells. (**A**) RNA fluorescence in situ hybridization (RNA-FISH) showing the spatial localization of lncRNA AP000654.1 (green) in mouse brain sections. Microglia were immunostained for Iba1 (red), and nuclei were counterstained with DAPI (blue). Representative images from negative control (NC) and lncRNA AP000654.1-overexpressing (OE) AAV-infected brains are shown. scale bar, 20 μm. (**B**) Western blot analysis and quantification of NLRP3 and cleaved caspase-1 p20 in HMC3 cells overexpressing lncRNA AP000654.1 (OE) or negative control (NC) under basal and OGD/R conditions (*n* = 3). (**C**) Western blot analysis and quantification of the pyroptosis executioner GSDMD-N and the pro-inflammatory cytokine IL-18 in HMC3 OE or NC cells under OGD/R conditions (*n* = 3). (**D**) Assessment of plasma membrane integrity by Hoechst 33,342/PI double staining. Representative images, scale bar, 100 μm. Quantification of PI mean fluorescence intensity (*n* = 5). LDH release was measured in culture supernatants of HMC3 OE or NC cells under OGD/R conditions (*n* = 3). *P < 0.05, **P < 0.01, ***P < 0.001

Long non-coding RNAs (lncRNAs) play key roles in cellular stress responses, such as those seen in cerebral ischemia. We previously reported that lncRNA AP000654.1 is downregulated in the peripheral blood of ischemic stroke patients, pointing to a role in disease. Here, we used OGD/R-treated HMC3 cells to investigate this further. OGD/R activated the NLRP3 inflammasome, pyroptosis, and mitophagy in these cells. RNA fluorescence in situ hybridization showed that lncRNA AP000654.1 is localized within microglial cells (Fig. 2A, Figure S2 A). Its expression was also significantly reduced after OGD/R (Figure S2 B). These findings suggest that lncRNA AP000654.1 is involved in the cellular stress response triggered by OGD/R.

To determine whether this reduction in lncRNA AP000654.1 has functional consequences, we examined its relationship with NLRP3 inflammasome activation following OGD/R. In wild-type HMC3 cells, OGD/R treatment increased NLRP3 protein levels compared to untreated controls (Control WT). We also observed elevations in cleaved caspase-1 (p20), GSDMD-N, and IL-18, though the increase in IL-18 did not reach statistical significance (Fig. 2B-C). These three proteins are markers of the pyroptotic pathway: p20 is the active form of caspase-1, GSDMD-N forms pores in the cell membrane, and IL-18 is a pro-inflammatory cytokine released upon inflammasome activation. Consistent with these molecular changes, OGD/R-treated cells showed increased plasma membrane permeability and elevated LDH release. LDH (lactate dehydrogenase) is a cytoplasmic enzyme; its presence in the culture medium indicates loss of membrane integrity, a hallmark of pyroptosis (Fig. 2D). Together, these results show that OGD/R induces functional NLRP3 inflammasome activation and downstream pyroptotic signaling in HMC3 cells.

We next examined whether restoring lncRNA AP000654.1 expression could mitigate the effects of OGD/R. In HMC3 cells, overexpressing this lncRNA reduced NLRP3 and cleaved caspase-1 (p20) protein levels compared to the negative control (NC) group under OGD/R conditions (Fig. 2B). Overexpression also decreased GSDMD-N, a marker of pyroptosis, and showed a trend toward reduced IL-18 (Fig. 2C). In addition, Hoechst 33,342/PI staining and LDH release assays indicated that AP000654.-overexpressing cells exhibited less membrane damage and fewer pyroptosis-associated changes than control cells after OGD/R (Fig. 2D). Notably, AP000654.1 overexpression under basal conditions did not significantly alter NLRP3 expression (Fig. 2B), suggesting that its regulatory effect becomes more evident under ischemic or inflammatory stress. Taken together, these findings support an inhibitory association between AP000654.1 expression and NLRP3 inflammasome-related pyroptotic responses in OGD/R-treated microglia.

Given that overexpressing lncRNA AP000654.1 reduces the release of pro-pyroptotic factors from OGD/R-treated microglia, we next examined whether this effect within microglia could send a paracrine signal to protect nearby neurons from pyroptosis.

### LncRNA AP000654.1 overexpression attenuates pyroptotic features in SH-SY5Y Neurons exposed to conditioned media from OGD/R-treated HMC3 Cells

Building upon the previous findings that lncRNA AP000654.1 suppresses NLRP3 inflammasome activation in microglial cells, we next investigated whether this regulation translates into indirect neuroprotection by altering the composition of the microglial secretome under ischemic conditions.

**Fig. 3:**
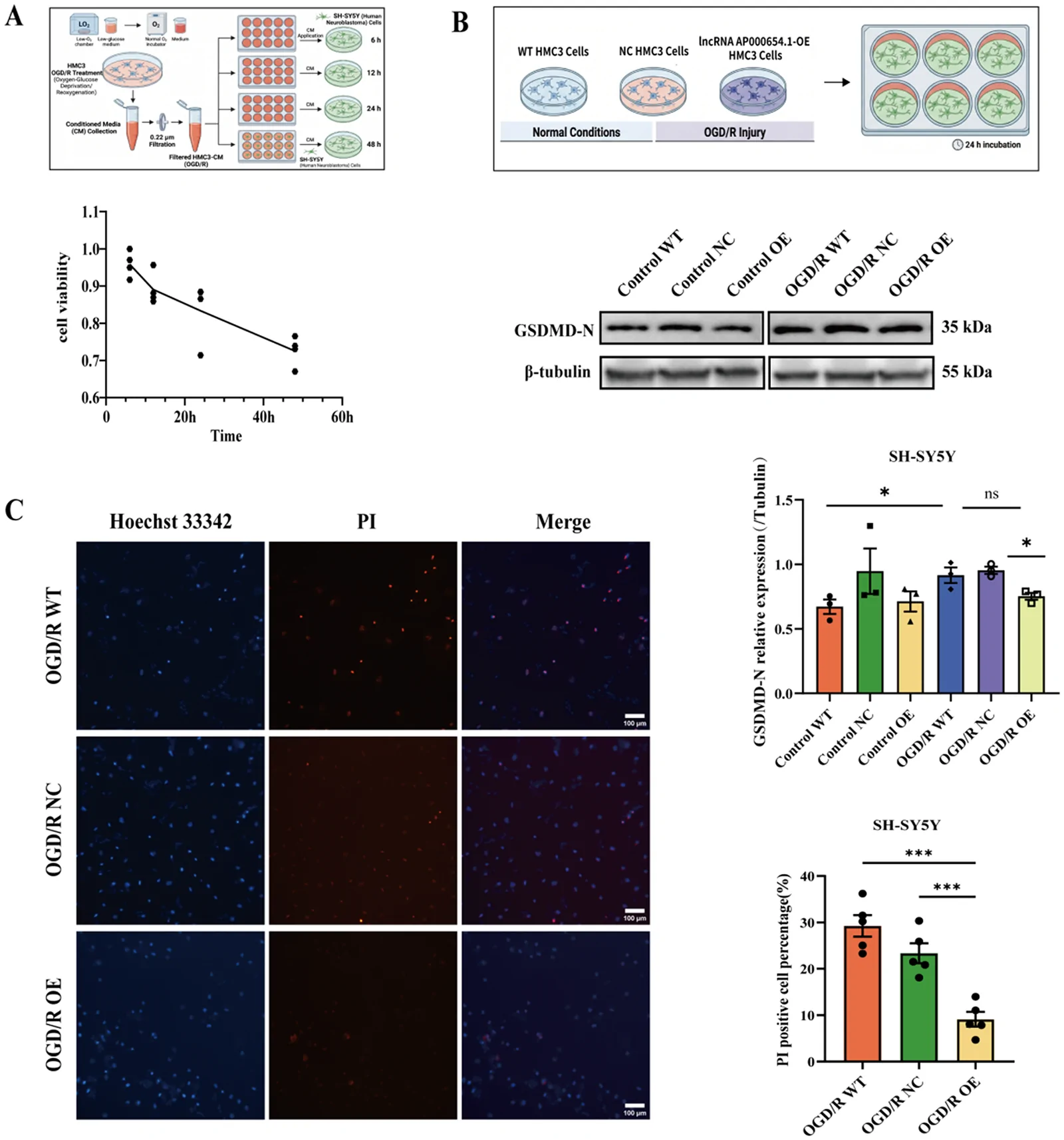
LncRNA AP000654.1 Overexpression in Microglia Reduces the Release of Pro-Pyroptotic Factors and Attenuates Pyroptotic Features in SH-SY5Y Neurons. (**A**) Time-course analysis of SH-SY5Y cell viability after exposure to conditioned medium (CM) collected from OGD/R-treated wild-type (WT) HMC3 microglia. Cell viability was measured by CCK-8 assay at the indicated time points. Based on this result, the 24 h time point was chosen for subsequent experiments (*n* = 3). (**B**) Western blot analysis of GSDMD-N in SH-SY5Y cells incubated for 24 h with CM from WT, negative control (NC), or lncRNA AP000654.1-overexpressing (OE) HMC3 cells cultured under either normal conditions or OGD/R stress (*n* = 3). (**C**) Hoechst 33,342/PI double staining to evaluate plasma membrane integrity in SH-SY5Y cells after 24 h incubation with CM from the indicated HMC3 groups. Representative images, scale bar, 100 μm. Quantification of PI mean fluorescence intensity (*n* = 5). *P < 0.05, **P < 0.01, ***P < 0.001

We first established a time-course model to determine the optimal incubation window. Conditioned media collected from OGD/R-injured wild-type HMC3 cells were applied to SH-SY5Y cells for 6 h, 12 h, 24 h, and 48 h. SH-SY5Y cell viability progressively declined with longer incubation times, indicating that OGD/R-injured microglia release detrimental factors that impair neuronal survival (Fig. 3A). Based on these results, the 24 h time point was selected for subsequent experiments. To further examine the role of lncRNA AP000654.1 in modulating microglia–neuron crosstalk, we collected conditioned media from six distinct HMC3 cell groups: wild-type HMC3 cells (WT), empty-vector control HMC3 cells (NC), and lncRNA AP000654.1-overexpressing HMC3 cells (OE) cultured under either normal conditions or subjected to OGD/R injury. These six groups were designed to address three specific questions: (1) whether OGD/R injury itself generates a pro-pyroptotic secretome (Control WT vs. OGD/R WT); (2) whether lentiviral transduction introduces confounding effects on secretome composition (OGD/R WT vs. OGD/R NC); and (3) whether AP000654.1 overexpression reduces the release of pyroptosis-inducing mediators under ischemic stress (OGD/R NC vs. OGD/R OE). SH-SY5Y cells incubated with conditioned media from OGD/R-injured WT or NC HMC3 cells exhibited markedly elevated GSDMD-N expression, indicating that OGD/R-stressed microglia release factors that trigger pyroptotic signaling in neurons. In contrast, SH-SY5Y cells treated with conditioned media from OGD/R-injured OE HMC3 cells showed reduced GSDMD-N expression compared to those receiving media from OGD/R-injured WT cells, suggesting that lncRNA AP000654.1 overexpression attenuates the release of pyroptosis-inducing factors from microglia under ischemic stress (Fig. 3B). Of note, no significant difference in GSDMD-N levels was observed among the three normal-condition groups, demonstrating that the protective effect of AP000654.1 is stress-dependent rather than constitutive.

Hoechst 33,342/PI double staining was performed on SH-SY5Y cells after 24 h incubation with each conditioned media. The proportion of PI-positive cells, reflecting plasma membrane permeabilization characteristic of pyroptosis, was significantly lower in the OGD/R OE group than in the OGD/R NC group (Fig. 3C).

Taken together, these data demonstrate that lncRNA AP000654.1 overexpression in microglia attenuates the release of pro-pyroptotic factors under OGD/R conditions, thereby reducing pyroptotic signaling in co-cultured neurons. These findings identify lncRNA AP000654.1 as a microglial regulatory node capable of modulating neuroinflammatory crosstalk during ischemic injury.

### LncRNA AP000654.1 alleviates OGD/R-induced injury by improving mitochondrial function and restraining excessive mitophagy

Given the observed neuroprotective effects described above, we investigated whether lncRNA AP000654.1 also preserves mitochondrial homeostasis in microglial cells under ischemia-reperfusion conditions, as mitochondrial dysfunction is a recognized upstream driver of NLRP3 inflammasome activation.

**Fig. 4:**
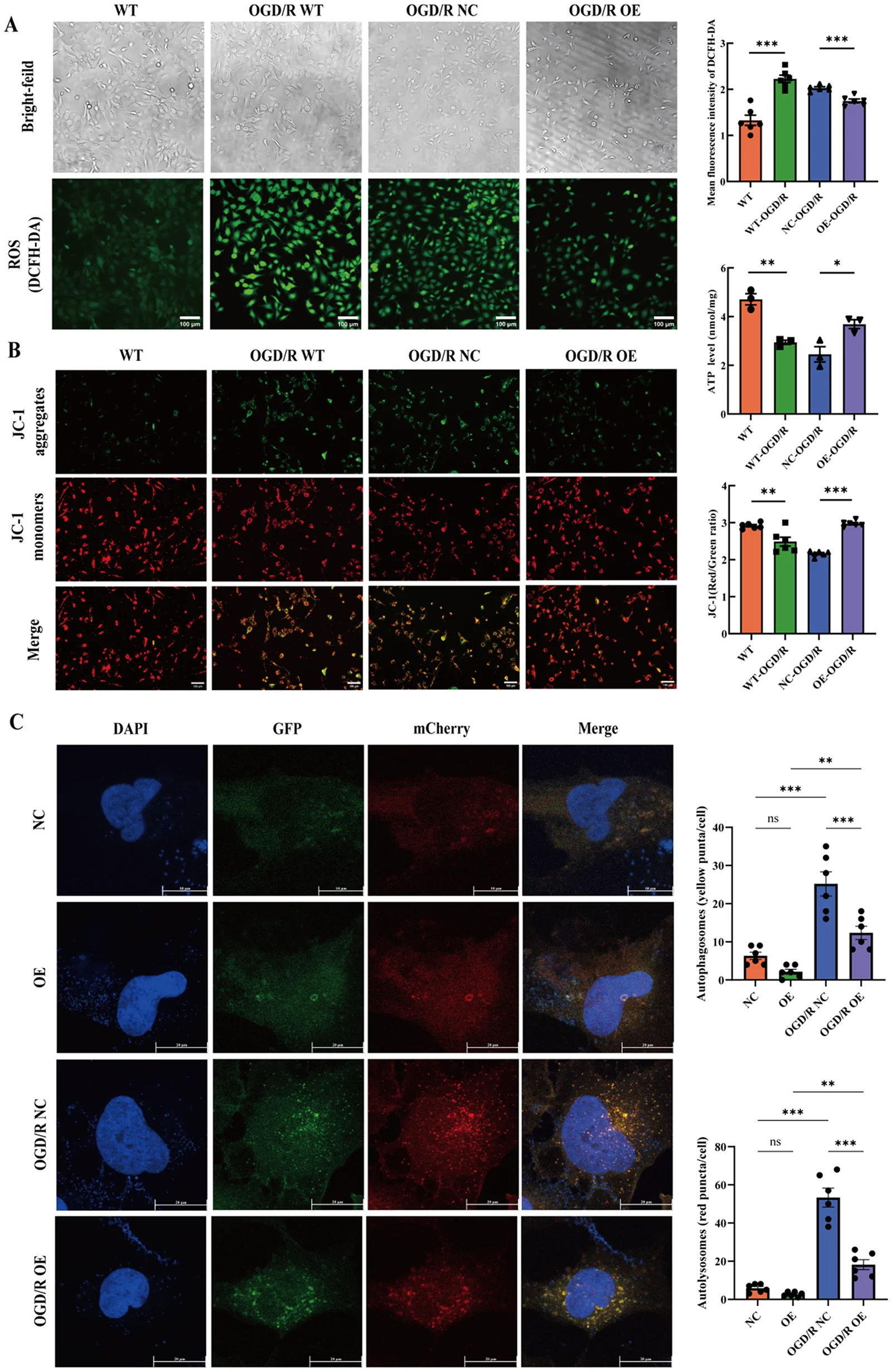
LncRNA AP000654.1 Alleviates OGD/R-Induced Mitochondrial Dysfunction and Modulates Autophagic Flux in HMC3 Microglia. (**A**) Intracellular reactive oxygen species (ROS) levels detected by DCFH-DA staining and fluorescence microscopy in OGD/R-treated negative control (NC) and AP000654.1-overexpressing (OE) HMC3 cells. Representative images, scale bar, 100 μm. Quantification of DCFH-DA mean fluorescence intensity is shown on the right (*n* = 6). Lower panel: cellular ATP levels measured by a bioluminescence assay in the same groups (*n* = 3). (**B**) Mitochondrial membrane potential (ΔΨm) assessed by JC-1 staining and fluorescence microscopy in OGD/R-treated NC and OE HMC3 cells. Representative images; scale bar, 100 μm. Quantification of JC-1 mean fluorescence intensity (green fluorescence, indicating mitochondrial depolarization) (*n* = 6). (**C**) Assessment of autophagic flux using the mRFP-GFP-LC3 adenoviral reporter. Representative confocal images of HMC3 cells from four groups: NC and OE cells under normal conditions, and NC and OE cells subjected to OGD/R. Yellow puncta (merged GFP and mCherry signals) indicate autophagosomes. OGD/R increased both autophagosome and autolysosome numbers in NC cells, whereas OE cells showed a relative reduction in puncta accumulation. Quantification of autophagosomes (yellow puncta per cell) and autolysosomes (red puncta per cell) is shown on the right (*n* = 6). Note that these measurements reflect general autophagic flux and do not directly quantify mitophagy-specific flux. Scale bar, 20 μm. *P < 0.05, **P < 0.01, ***P < 0.001

Wild-type (WT), negative control (NC), and lncRNA AP000654.1-overexpressing (OE) HMC3 cells were subjected to OGD/R treatment. Intracellular reactive oxygen species (ROS) production was measured using the DCFH-DA probe, and cellular ATP levels were quantified with a luminescence-based assay kit. As shown in Fig. 4A and B, OE cells exhibited significantly lower ROS accumulation and higher ATP production relative to NC cells under OGD/R conditions, indicating that AP000654.1 overexpression mitigates mitochondrial dysfunction induced by ischemia-reperfusion.

We next examined whether the mitochondrial alterations induced by OGD/R were accompanied by changes in mitophagy-related flux. Because excessive mitochondrial turnover under pathological stress may deplete functional mitochondria, impair energy metabolism, and aggravate inflammatory signaling, we evaluated autophagic flux using the mRFP-GFP-LC3 reporter system. In NC cells exposed to OGD/R, abundant yellow and red puncta were observed in the cytoplasm, indicating increased autophagosome formation and autolysosome maturation. In contrast, AP000654.1-overexpressing cells displayed a lower puncta burden and a partial normalization of puncta distribution (Fig. 4C). These findings suggest that AP000654.1 attenuates the excessive autophagy-related flux induced by OGD/R.

**Fig. 5:**
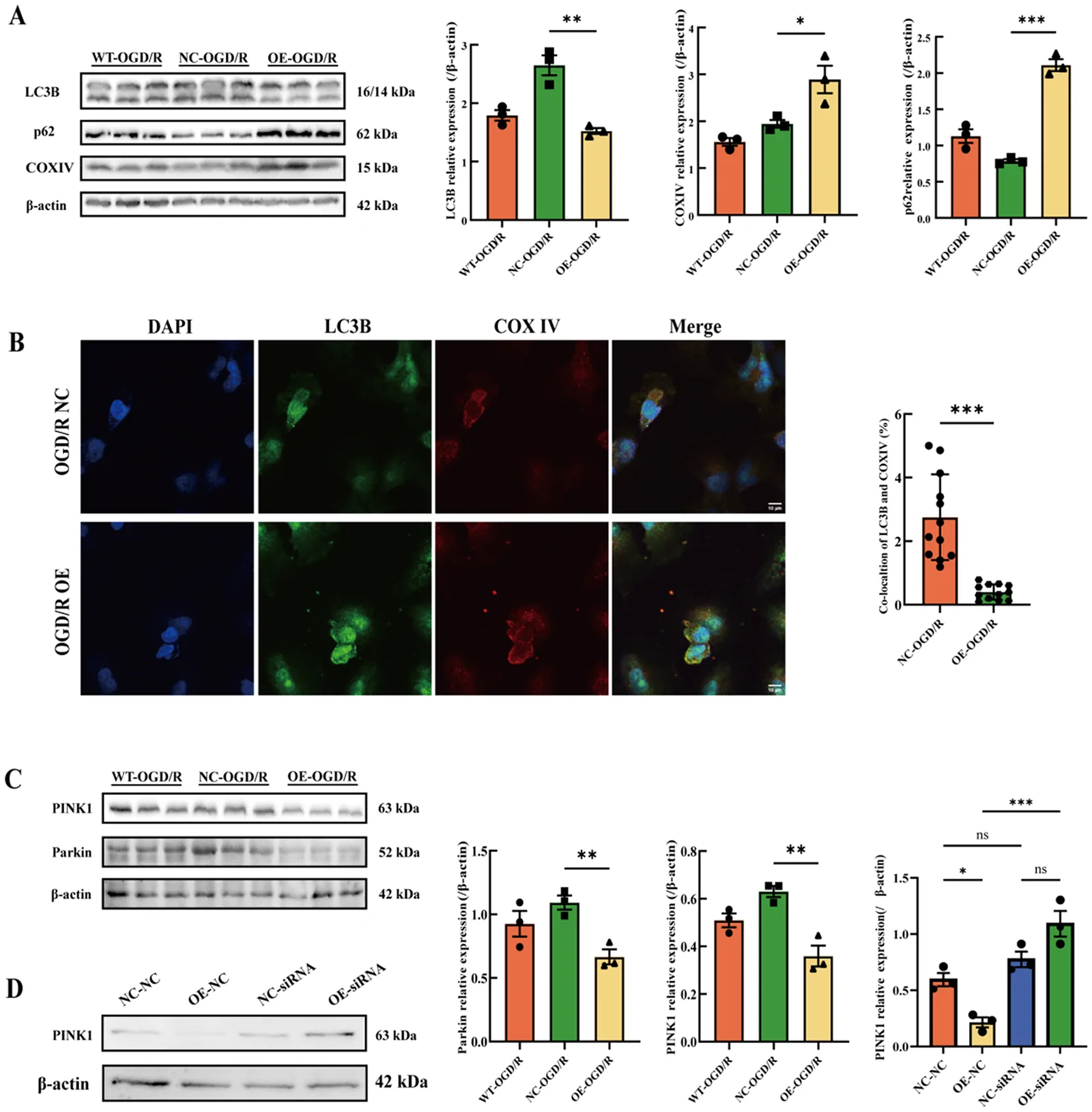
LncRNA AP000654.1 Overexpression Attenuates OGD/R-Induced Autophagy-Related and Mitophagy-Associated Marker Changes in HMC3 Cells, While Its Knockdown Exerts Opposite Effects. (**A**) Western blot analysis and quantification of the general autophagy markers LC3B (LC3B-II) and p62/SQSTM1, and the mitochondrial protein COXIV, in negative control (NC) and AP000654.1-overexpressing (OE) HMC3 cells subjected to OGD/R (*n* = 3). (**B**) Representative immunofluorescence images showing colocalization of LC3B (green) and the mitochondrial protein COXIV (red) in post-OGD/R NC and OE HMC3 cells. Nuclei were counterstained with DAPI (blue). Scale bar, 10 μm. Quantification of LC3B–COXIV colocalization is shown on the right (*n* = 12 cells). Note that LC3B–COXIV colocalization provides indirect evidence for mitochondria-associated autophagic activity but does not directly measure mitophagy-specific flux. (**C**) Western blot analysis and quantification of PINK1 and Parkin protein levels in OGD/R-treated NC and OE HMC3 cells (*n* = 3). (**D**) Western blot analysis and quantification of PINK1 protein expression in HMC3 cells following OGD/R treatment and subsequent knockdown of lncRNA AP000654.1 using siRNA (*n* = 3). *P < 0.05, **P < 0.01, ***P < 0.001

When interpreted together with the mitochondrial marker changes described below, these data are consistent with suppression of excessive mitophagy-associated activity rather than a complete blockade of basal autophagy. Consistent with this interpretation, AP000654.1-overexpressing cells showed lower mitochondrial ROS levels and higher ATP production than NC cells under OGD/R conditions. These findings indicate that AP000654.1 helps preserve mitochondrial integrity under ischemic stress. We then evaluated mitophagy-associated mitochondrial turnover by examining LC3B, P62, and COXIV expression. In OGD/R-treated cells, AP000654.1 overexpression reduced LC3B levels while increasing P62 and COXIV expression relative to control cells (Fig. 5A). By contrast, AP000654.1 knockdown produced the opposite pattern, with increased LC3B and decreased P62 expression (Figure S3). Although LC3B and P62 are not specific markers of mitophagy, the combined changes in LC3B/P62 together with restoration of the mitochondrial protein COXIV support the interpretation that AP000654.1 limits excessive mitochondria-associated autophagic turnover under OGD/R conditions. Immunofluorescence analysis further showed that colocalization of LC3B and COXIV was reduced in AP000654.1-overexpressing cells (Fig. 5B), providing additional support for decreased sequestration of mitochondrial material by autophagic structures.

We next examined whether this effect was associated with the PINK1-Parkin pathway. When mitochondria lose membrane potential, PINK1 stabilizes on the outer mitochondrial membrane and recruits Parkin, an E3 ubiquitin ligase, to tag damaged mitochondria for autophagic clearance. After OGD/R, AP000654.1-overexpressing cells showed a decreasing trend in PINK1 expression and a statistically significant reduction in Parkin levels compared to NC cells (Fig. 5C). Conversely, knocking down AP000654.1 increased PINK1 protein levels (Fig. 5D). These findings suggest that AP000654.1 is associated with modulation of PINK1–Parkin pathway associated mitophagy signaling; however, they do not by themselves establish a direct mechanistic interaction between AP000654.1 and the PINK1–Parkin pathway.

### Excessive mitophagy aggravates NLRP3 inflammasome activation and pyroptosis, which is attenuated by lncRNA AP000654.1

To further assess the relationship between mitophagy-associated mitochondrial turnover and NLRP3 inflammasome activation under ischemic stress, we used rapamycin (RAPA), an mTOR inhibitor that enhances autophagy and has been widely used to promote mitophagy-related responses.

**Fig. 6:**
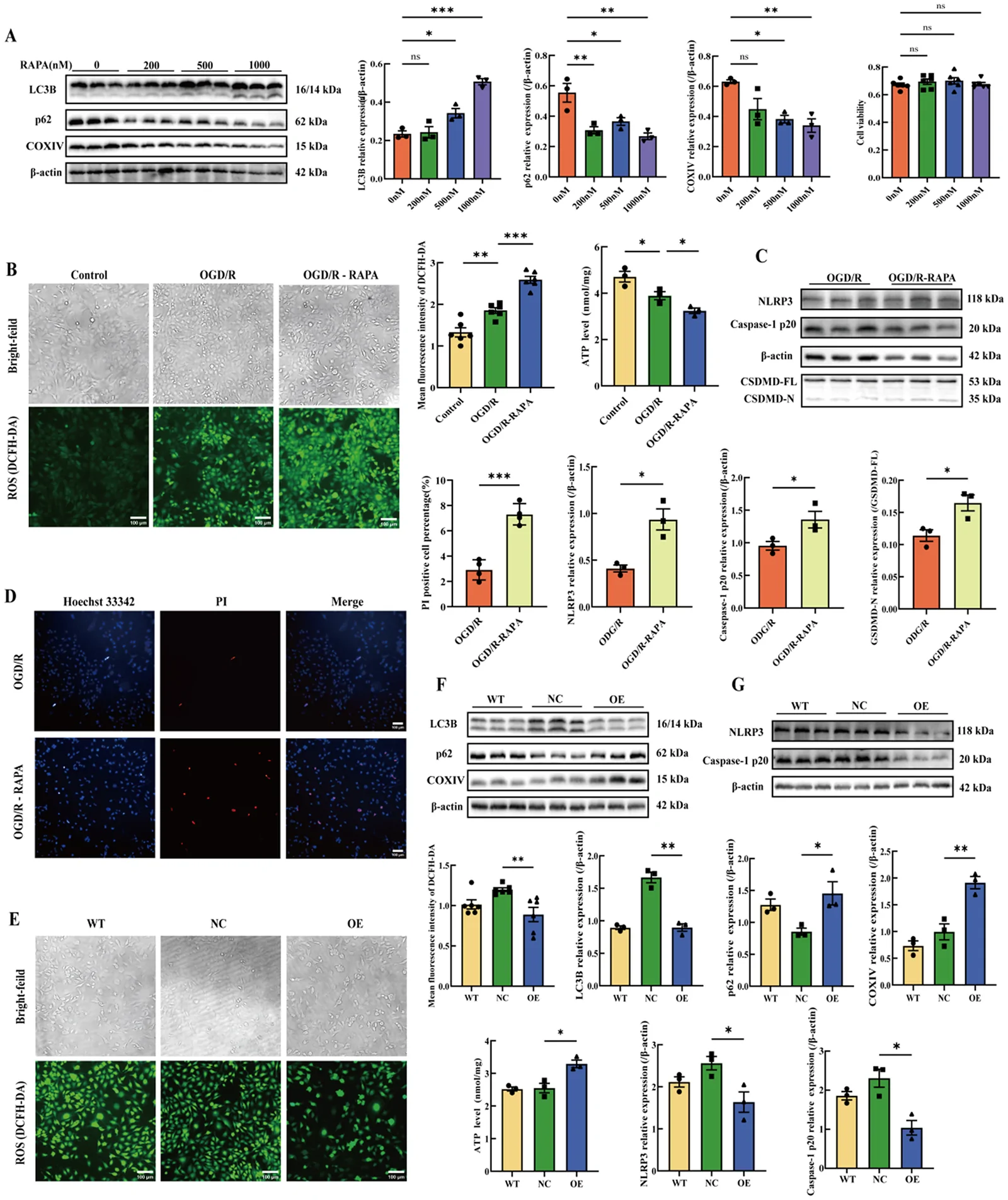
Pharmacological Enhancement of Autophagy/Mitophagy-Associated Turnover by Rapamycin Aggravates OGD/R-Induced NLRP3 Inflammasome Activation and Pyroptosis, Which Is Attenuated by LncRNA AP000654.1 Overexpression. (**A**) Western blot analysis and quantification of LC3B (LC3B-II), p62/SQSTM1, and COXIV in HMC3 cells treated with rapamycin (RAPA, 1000 nM) for 24 h (*n* = 3), confirming effective induction of general autophagy and associated mitochondrial turnover. (**B**) Intracellular ROS levels detected by DCFH-DA staining and fluorescence microscopy in HMC3 cells subjected to OGD/R with or without RAPA pretreatment. Representative images; scale bar, 100 μm. Quantification of DCFH-DA mean fluorescence intensity (*n* = 6). Cellular ATP levels measured by a bioluminescence assay in the same groups (*n* = 3). (**C**) Western blot analysis and quantification of NLRP3, cleaved caspase-1 p20, and GSDMD-N expression in HMC3 cells subjected to OGD/R alone or OGD/R combined with RAPA pretreatment (OGD/R+RAPA) (*n* = 3). (**D**) Hoechst 33,342/PI double staining to evaluate plasma membrane integrity in HMC3 cells treated with OGD/R alone or OGD/R+RAPA. Representative images; scale bar, 100 μm. Quantification of PI mean fluorescence intensity (*n* = 4). (**E**) DCFH-DA staining for ROS in wild-type (WT), negative control (NC), and AP000654.1-overexpressing (OE) HMC3 cells pretreated with RAPA and subjected to OGD/R. Representative images; scale bar, 100 μm. Quantification of DCFH-DA mean fluorescence intensity (*n* = 6). (**F**) Western blot analysis and quantification of LC3B, p62, and COXIV in WT, NC, and OE HMC3 cells pretreated with RAPA and subjected to OGD/R (*n* = 3). (**G**) Western blot analysis and quantification of NLRP3 and cleaved caspase-1 p20 in WT, NC, and OE HMC3 cells pretreated with RAPA and subjected to OGD/R (*n* = 3). *P < 0.05, **P < 0.01, ***P < 0.001

Dose-optimization experiments showed that 1000 nM RAPA effectively increased LC3B and reduced P62 and COXIV expression without significantly compromising cell viability (Fig. 6A). Under OGD/R stress, RAPA-treated cells exhibited higher ROS levels and lower ATP production than untreated OGD/R cells (Fig. 6B), indicating aggravated mitochondrial dysfunction. Moreover, RAPA treatment was also accompanied by increased NLRP3, cleaved Caspase-1 (p20), and GSDMD-N (Fig. 6C), together with an increased proportion of PI-positive cells (Fig. 6D). These data support the view that excessive autophagy/mitochondria-associated turnover under OGD/R is linked to enhanced inflammasome-related injury.To further evaluate the regulatory effect of AP000654.1 in this context, wild-type, negative control, and AP000654.1-overexpressing HMC3 cells were pretreated with RAPA before OGD/R exposure. AP000654.1 overexpression reduced ROS accumulation and partially restored ATP levels compared with control cells (Fig. 6E). Western blot analysis showed lower LC3B expression together with relative restoration of COXIV levels in AP000654.1-overexpressing cells (Fig. 6F), supporting a reduction in excessive mitochondria-associated autophagic turnover. In parallel, AP000654.1 overexpression attenuated the RAPA-associated increases in NLRP3 and cleaved caspase-1 p20 expression (Fig. 6G). These findings are consistent with the possibility that the anti-inflammatory effect of AP000654.1 is linked, at least in part, to limiting excessive mitophagy-associated stress under OGD/R conditions.

## Discussion

Ischemic stroke involves complex crosstalk between mitochondrial dysfunction and neuroinflammation, yet the endogenous regulators linking these processes remain incompletely understood. In the present study, our data support an association between lncRNA AP000654.1 and attenuation of mitophagy-associated mitochondrial stress and NLRP3 inflammasome-associated pyroptotic responses in microglia. Under OGD/R stress, AP000654.1 overexpression was accompanied by reduced inflammatory activation, lower mitochondrial ROS accumulation, improved ATP production, and altered expression of PINK1–Parkin-related proteins together with changes in general autophagy-associated markers. These findings suggest that AP000654.1 may help limit a pathological feed-forward cycle between mitochondrial injury and inflammation.

Bioinformatic analysis and RNA-FISH suggested that AP000654.1 is predominantly expressed in microglia, supporting a potential role in neuroinflammatory regulation. Functionally, AP000654.1 overexpression reduced OGD/R-induced NLRP3 inflammasome-associated changes, including decreased NLRP3 and cleaved caspase-1 p20 expression, along with lower ROS levels, improved ATP production, and partial normalization of autophagy-related markers. These observations are consistent with a role for AP000654.1 in attenuating NLRP3-driven inflammatory responses under ischemic conditions, although it should be noted that the autophagy-related markers measured here reflect general autophagic activity rather than mitophagy-specific flux.

The importance of NLRP3 inflammasome activation in ischemic stroke has been established by multiple studies. Li et al. reported increased NLRP3, ASC, caspase-1, and IL-1β expression in MCAO models [14]. Wang et al. confirmed robust NLRP3 activation in OGD/R-treated neuronal cells [32]. Ge et al. showed that TMAO activates microglial NLRP3 under OGD/R conditions, thereby exacerbating ischemic injury [33]. Franke et al. found that NLRP3 mRNA increased more than 20-fold in ischemic hemispheres after tMCAO, and that treatment with an NLRP3 inhibitor reduced infarct volume and caspase-1 activation [34]. Collectively, these studies indicate that excessive NLRP3 inflammasome activation contributes to ischemic stroke pathology and underscore the need to identify upstream regulatory mechanisms.

Based on these observations, we hypothesized that the anti-inflammatory effect of AP000654.1 may be related, at least in part, to regulation of mitochondrial dysfunction. Mitochondrial injury is a well-recognized trigger for NLRP3 inflammasome activation, and our results are consistent with this framework. In the present study, AP000654.1 overexpression was accompanied by reduced ROS accumulation, preserved ATP production, and partial restoration of mitochondrial homeostasis under OGD/R conditions. These findings support a functional association between AP000654.1 and the maintenance of mitochondrial integrity.

We further considered whether the effects of AP000654.1 might be related to the PINK1–Parkin pathway, a central regulator of mitochondrial quality control. AP000654.1 overexpression was accompanied by reduced Parkin expression and a decreasing trend in PINK1 levels after OGD/R, whereas AP000654.1 knockdown showed the opposite tendency. These results are consistent with an association between AP000654.1 and the PINK1–Parkin pathway. However, because the present study did not directly examine molecular binding, transcriptional regulation, or pathway-specific rescue involving PINK1 or Parkin, this relationship should be interpreted as associative rather than definitively mechanistic.

Our rapamycin experiments provided additional evidence linking enhanced autophagy/mitochondria-associated turnover with inflammatory injury under OGD/R conditions. RAPA treatment aggravated ROS accumulation, ATP depletion, and NLRP3 inflammasome-related responses, whereas AP000654.1 overexpression partially attenuated these effects. Because rapamycin is a general autophagy activator rather than a mitophagy-specific agent, these findings cannot be attributed exclusively to mitophagy modulation. Nevertheless, when considered alongside the autophagic flux and mitochondrial marker data, these results provide supportive—though not definitive—evidence that AP000654.1 may modulate mitophagy-associated processes under ischemic stress conditions.

The present findings are therefore more consistent with a role for AP000654.1 in preserving mitochondrial homeostasis under ischemic stress than with a model in which AP000654.1 completely suppresses mitophagy. However, given that RAPA broadly activates autophagy and that mitophagy-specific flux was not directly measured, any conclusion regarding selective mitochondrial elimination as the predominant mechanism remains premature.

The role of mitophagy in disease is highly context dependent, and this distinction between basal protective quality control and detrimental excessive turnover is supported by previous studies. Qiu et al. reported that AMPK-mediated autophagy reduces ischemic injury by suppressing inflammasome activation [35]. In contrast, Dupont et al. showed that excessive autophagy can promote caspase-1 activation and IL-1β secretion [36]. Under severe OGD/R stress, the PINK1–Parkin pathway may become excessively activated, leading to disproportionate mitochondrial turnover and impaired mitochondrial homeostasis. In this setting, mitochondria-derived signals—including cytosolic mtDNA, which can act as a NLRP3 agonist, and elevated mitochondrial ROS, which directly facilitates NLRP3 complex assembly—may together amplify inflammatory signaling beyond what cellular quality-control systems can resolve. Within this framework, AP000654.1 may function as a homeostatic regulator that helps prevent mitochondrial turnover-related stress from shifting toward a pro-inflammatory state.

LncRNAs are increasingly recognized as important regulators of neuroinflammation and autophagy-related processes. Yao et al. showed that silencing lncRNA TUG1 inhibits NLRP3 activation and pyroptosis [27]. Deng et al. found that SNHG14 promotes mitophagy and apoptosis via the miR-182-5p/BNIP3 axis under OGD/R conditions [37]. Our study extends this line of evidence by suggesting that AP000654.1 is associated with the crosstalk between mitochondria-associated autophagic stress and inflammasome activation. Together with prior reports on TUG1 and SNHG14, these findings are consistent with a model in which individual lncRNAs regulate distinct nodes within the mitochondrial stress–inflammasome axis, collectively suggesting a multi-level regulatory network that may offer multiple potential therapeutic entry points.

This study has several limitations. First, the functional assays were performed in vitro using HMC3 cells, which do not fully recapitulate the complexity of ischemic injury in vivo. Validation in animal models, such as MCAO mice, will therefore be necessary. Second, although we focused on the PINK1–Parkin pathway, other mitophagy-related regulators, including BNIP3 and FUNDC1, may also contribute to the function of AP000654.1 and should be investigated in future studies. Third, although autophagic flux was assessed using the mRFP-GFP-LC3 reporter system, mitophagy-specific flux was not directly quantified; future experiments employing mitophagy-specific reporters, such as mt-Keima or mito-QC, would provide more direct evidence for selective mitochondrial elimination. Fourth, direct mechanistic validation linking AP000654.1 to the PINK1–Parkin pathway—such as Parkin mitochondrial translocation assays, RNA immunoprecipitation analysis, or pathway-specific rescue experiments with PINK1/Parkin modulation—was not performed in the current study; these experiments are planned and will be incorporated into our follow-up work. Fifth, bioinformatic analysis has identified a promising candidate as a potential downstream target of AP000654.1, and preliminary data support this interaction. A dedicated mechanistic investigation is currently underway and will be reported in a subsequent publication.

Our findings suggest that lncRNA AP000654.1 is associated with suppression of excessive mitophagy-associated mitochondrial stress and NLRP3 inflammasome activation under ischemic conditions. By preserving mitochondrial homeostasis and attenuating inflammatory responses, AP000654.1 may help protect microglia from OGD/R-induced injury under these experimental conditions. These results provide preliminary evidence that lncRNA-mediated modulation of mitochondria-associated autophagic stress may converge on inflammasome signaling in the context of cerebral ischemia-reperfusion injury, and suggest that AP000654.1 warrants further mechanistic and in vivo investigation as a potential therapeutic target.

## Supplementary Information

Below is the link to the electronic supplementary material.

Supplementary Material 1: Figure S1. Time-Dependent Induction of NLRP3 mRNA During OGD in HMC3 Microglia. Quantitative RT-PCR analysis of NLRP3 mRNA levels in HMC3 cells exposed to OGD for the indicated durations (0, 2, 4, 6, and 8 h) (*n* = 3). **P < 0.01, ***P < 0.001. Figure S2. Neuronal Localization and OGD-Responsive Downregulation of LncRNA AP000654.1. (A) RNA-FISH detection of lncRNA AP000654.1 (green) in neurons of mouse brain sections. Neurons were labeled with NeuN (red) and nuclei with DAPI (blue). NC, negative control AAV; OE, lncRNA AP000654.1-overexpressing AAV. scale bar, 20 µm. (B) Quantitative RT-PCR analysis of lncRNA AP000654.1 expression in HMC3 cells exposed to OGD for the indicated durations. Expression was significantly reduced during OGD (*n* = 3). *P < 0.05, **P < 0.01. Figure S3. Knockdown of LncRNA AP000654.1 Promotes OGD/R-Induced NLRP3 Inflammasome Activation, Pyroptosis Marker Expression, and Autophagy-Related Changes. Western blot analysis and quantification of NLRP3, p62, Beclin1, GSDMD-N, Caspase‑1 p20, and LC3B protein expression in HMC3 cells following OGD/R treatment and siRNA‑mediated knockdown of lncRNA AP000654.1 (*n* = 3). *P < 0.05, **P < 0.01, ***P < 0.001.

## Data Availability

All data generated or analyzed during this study are included in this published article and its supplementary information files.The data that support the findings of this study are available from the corresponding author upon reasonable request.

## Abbreviations

IS: Ischemic stroke
DAMPs: Damage-associated molecular patterns
PRRs: Pattern recognition receptors
mtROS: Mitochondrial reactive oxygen species
mtDNA: Mitochondrial DNA
lncRNAs: Long non-coding RNAs
OGD/R: Deprivation/reoxygenation
RAPA: Rapamycin
PVDF: Polyvinylidene difluoride
I/R: Ischemia-reperfusion
