# Supplementary material for: LncRNA AP000654.1 attenuates cerebral ischemia-reperfusion injury by regulating mitophagy-mediated NLRP3 inflammasome activation
Source: J Transl Med. 2026 Jul 7;24:1230. doi: 10.1186/s12967-026-08541-z (PMC13628737; doi:10.1186/s12967-026-08541-z)

Figure 1E

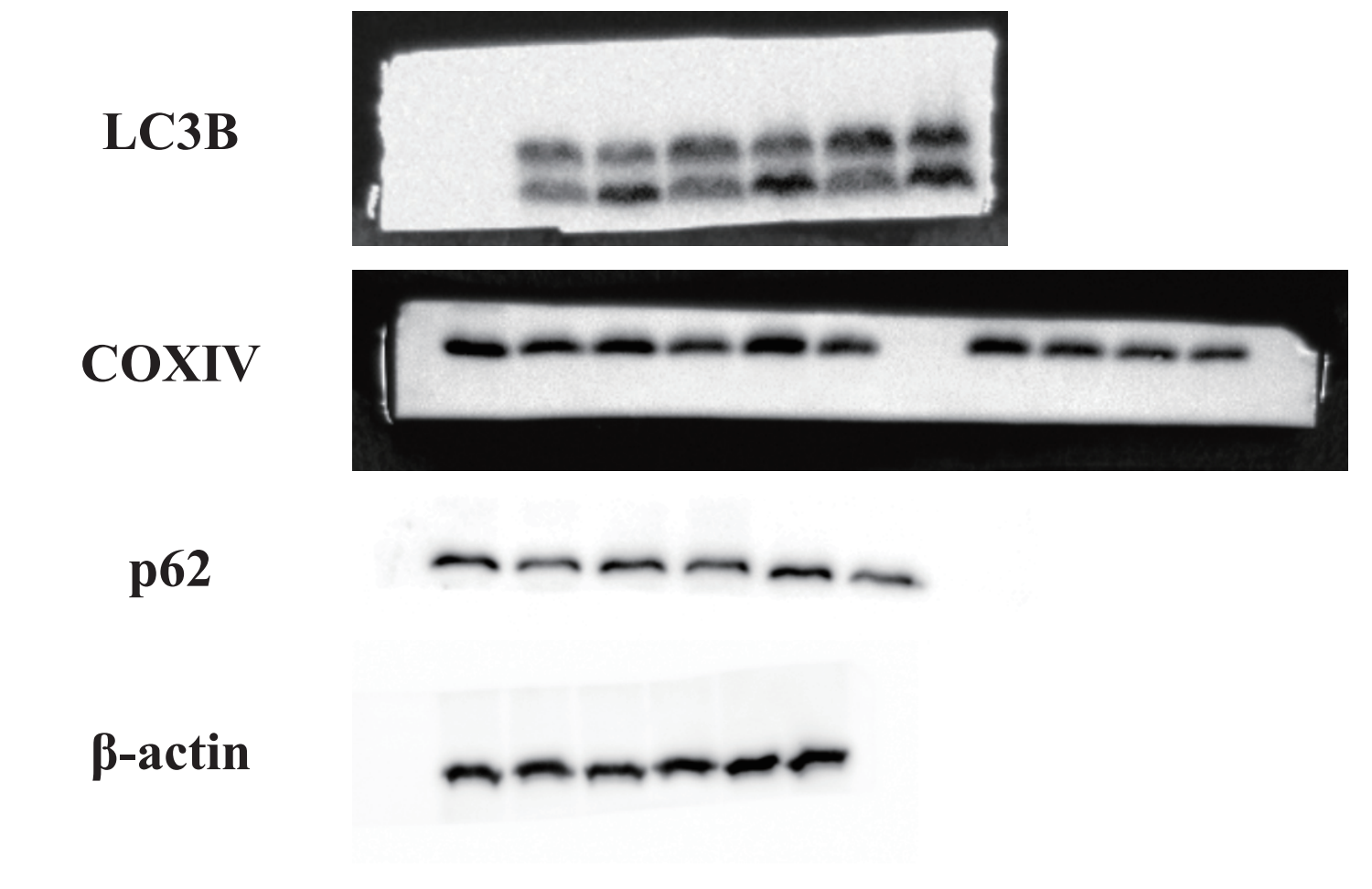

Figure 2B

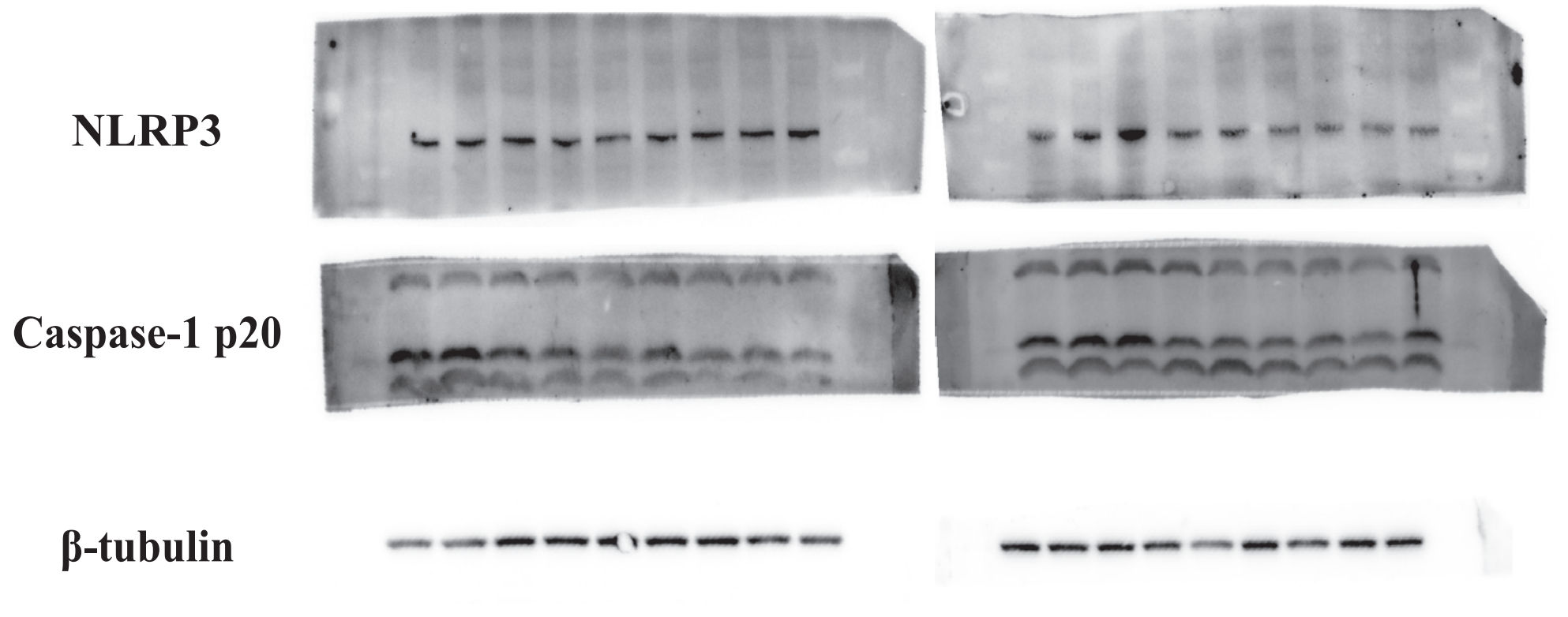

Figure 2C

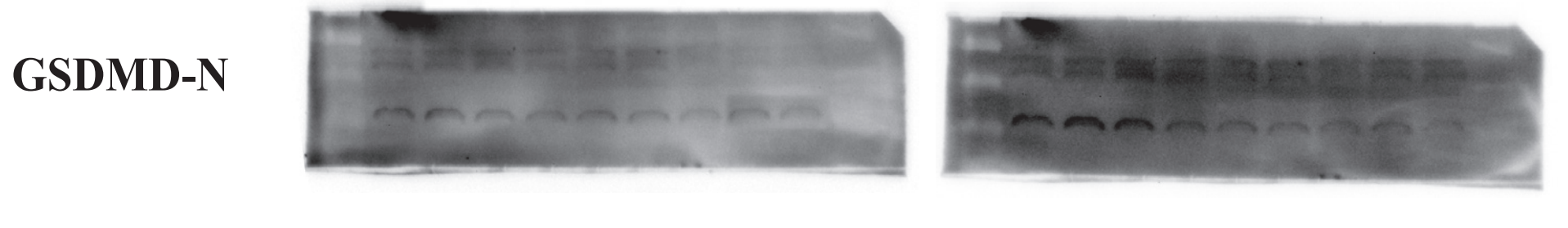

Figure 3B

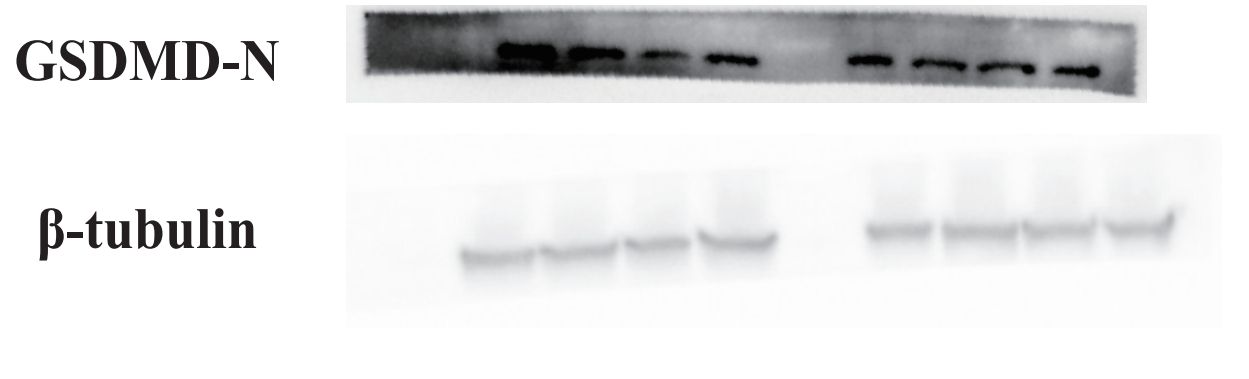

Figure 5A

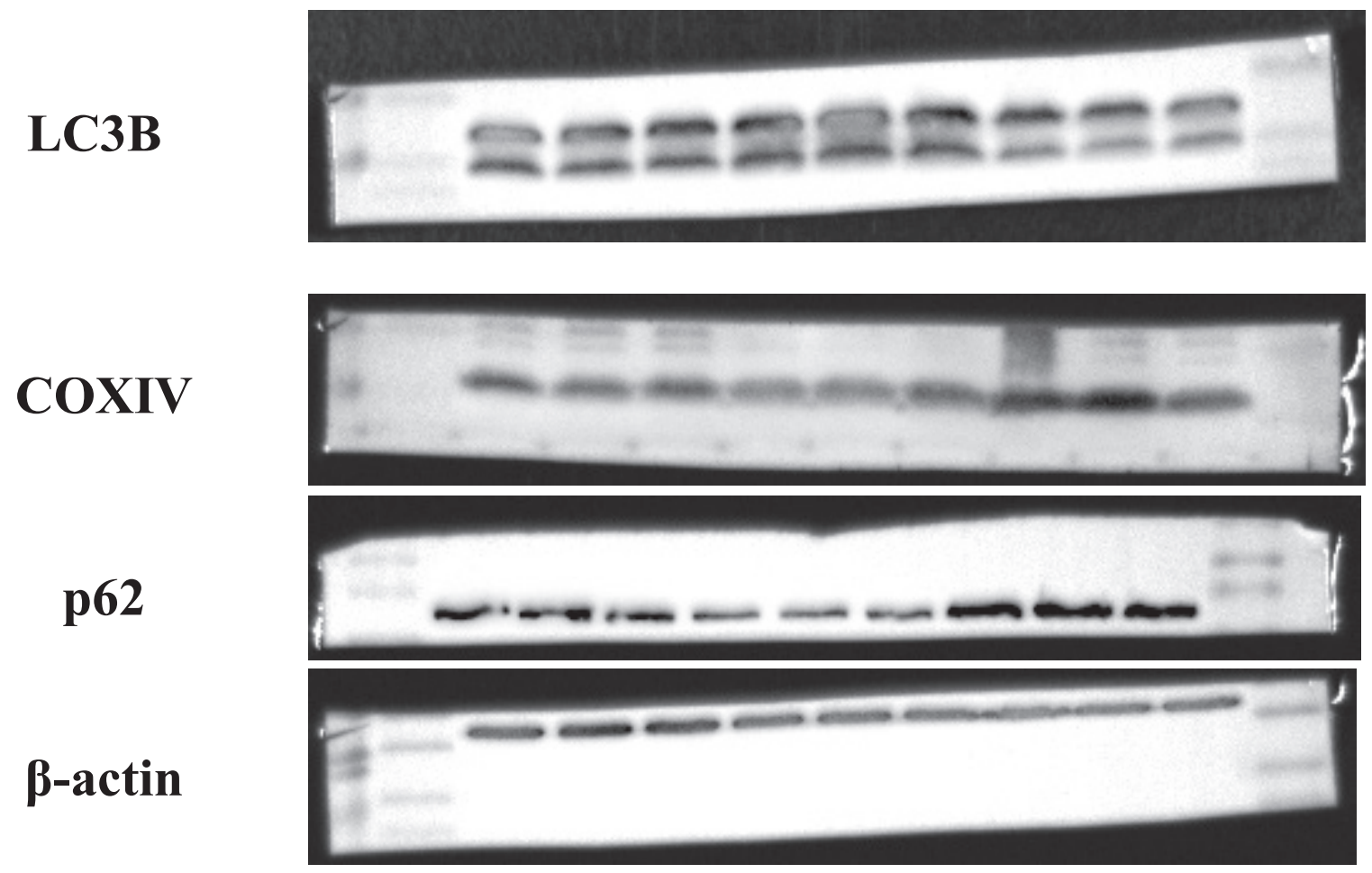

Figure 5C

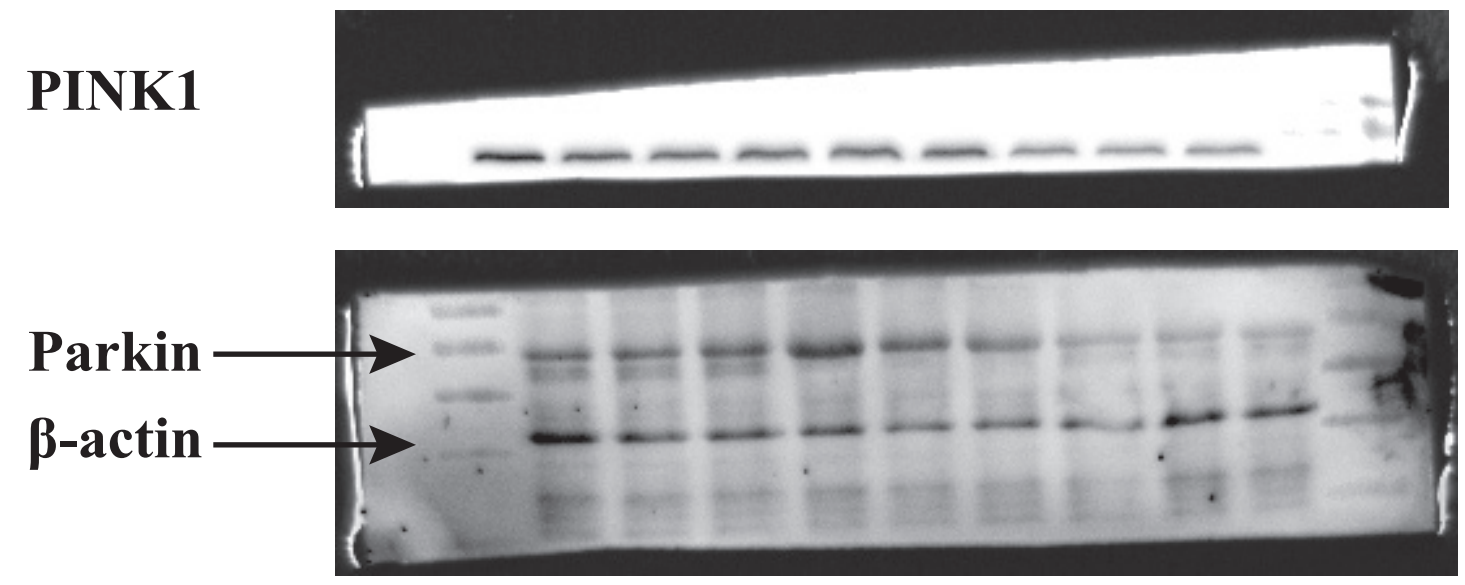

Figure 5D

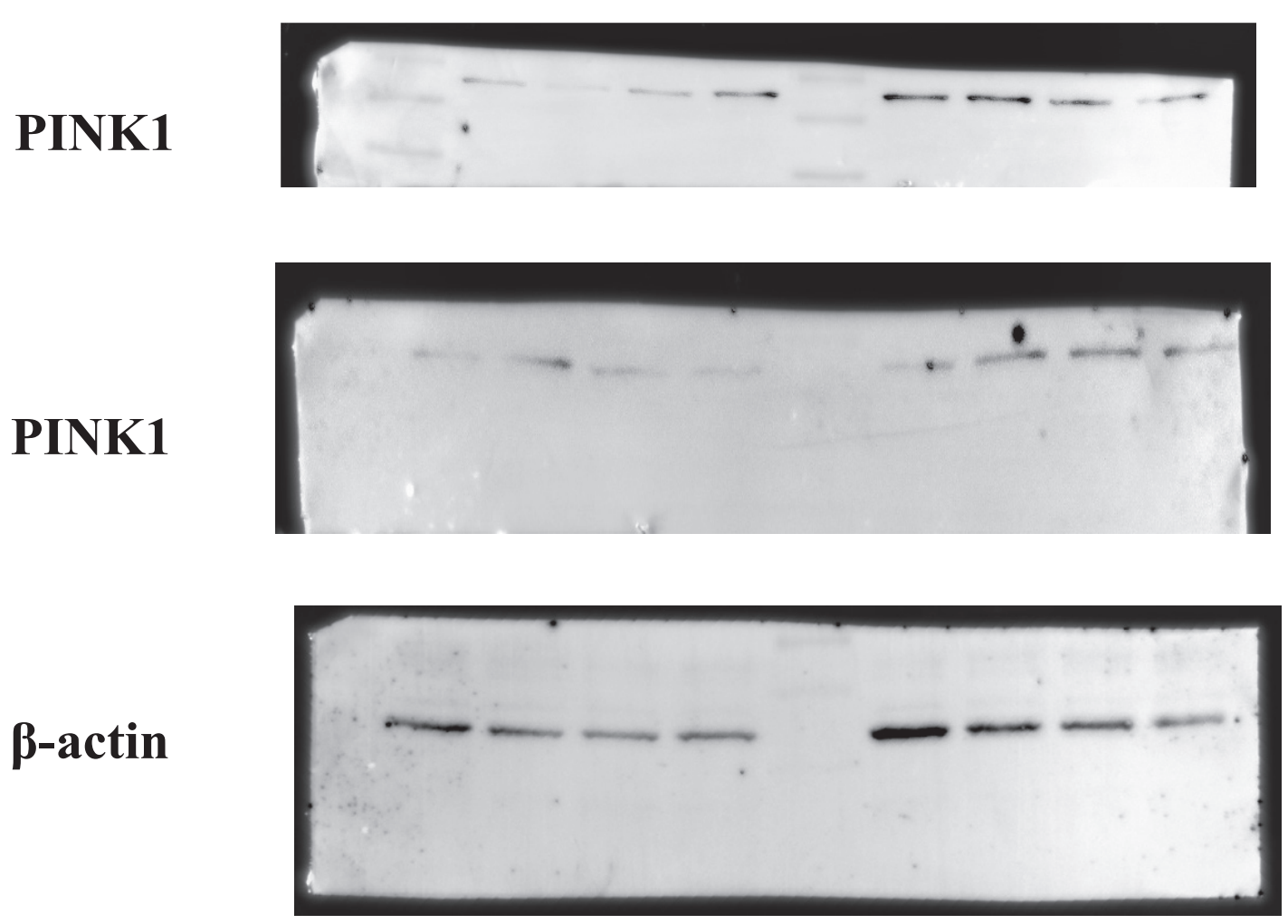

Figure 6A

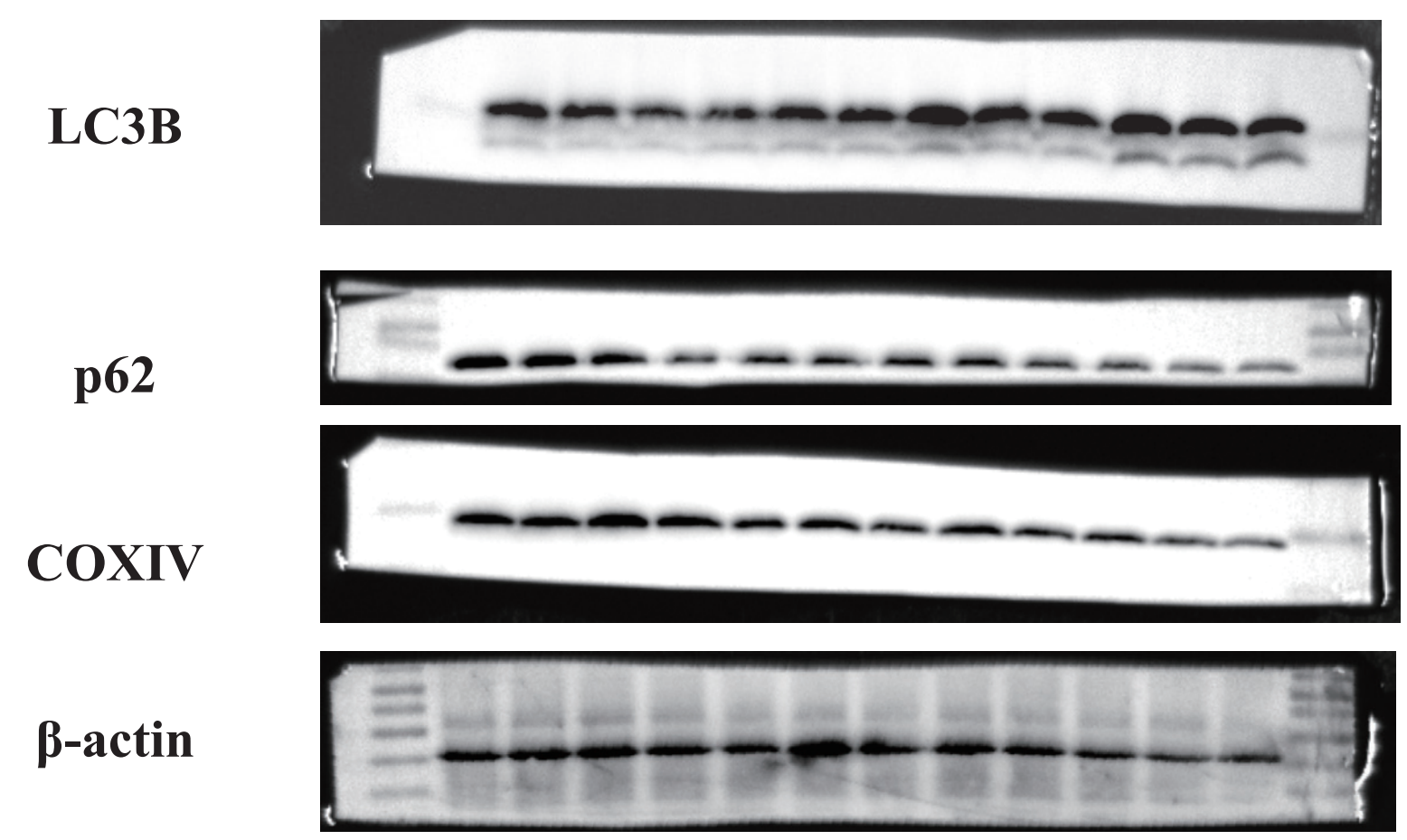

Figure 6C

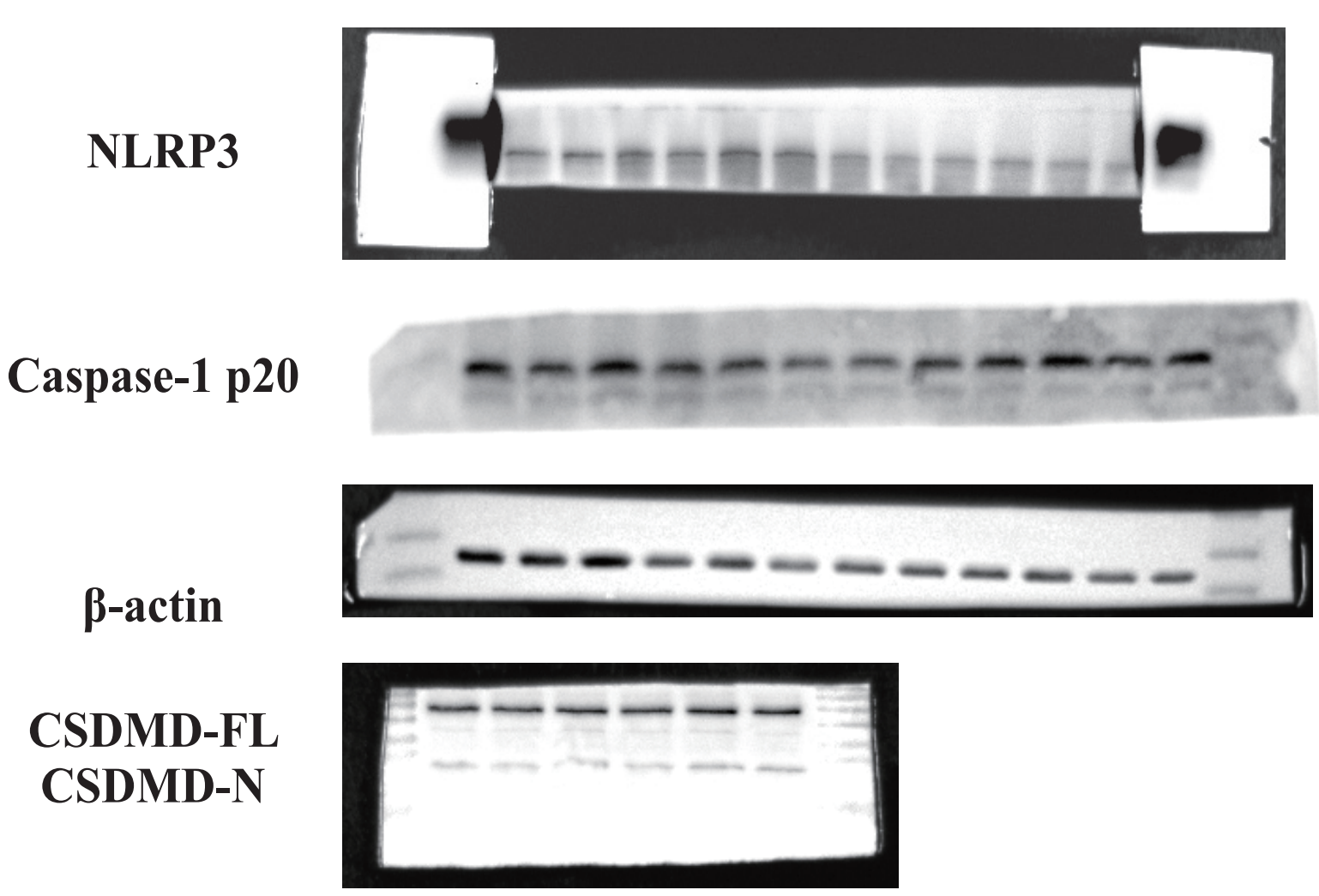

Figure 6F

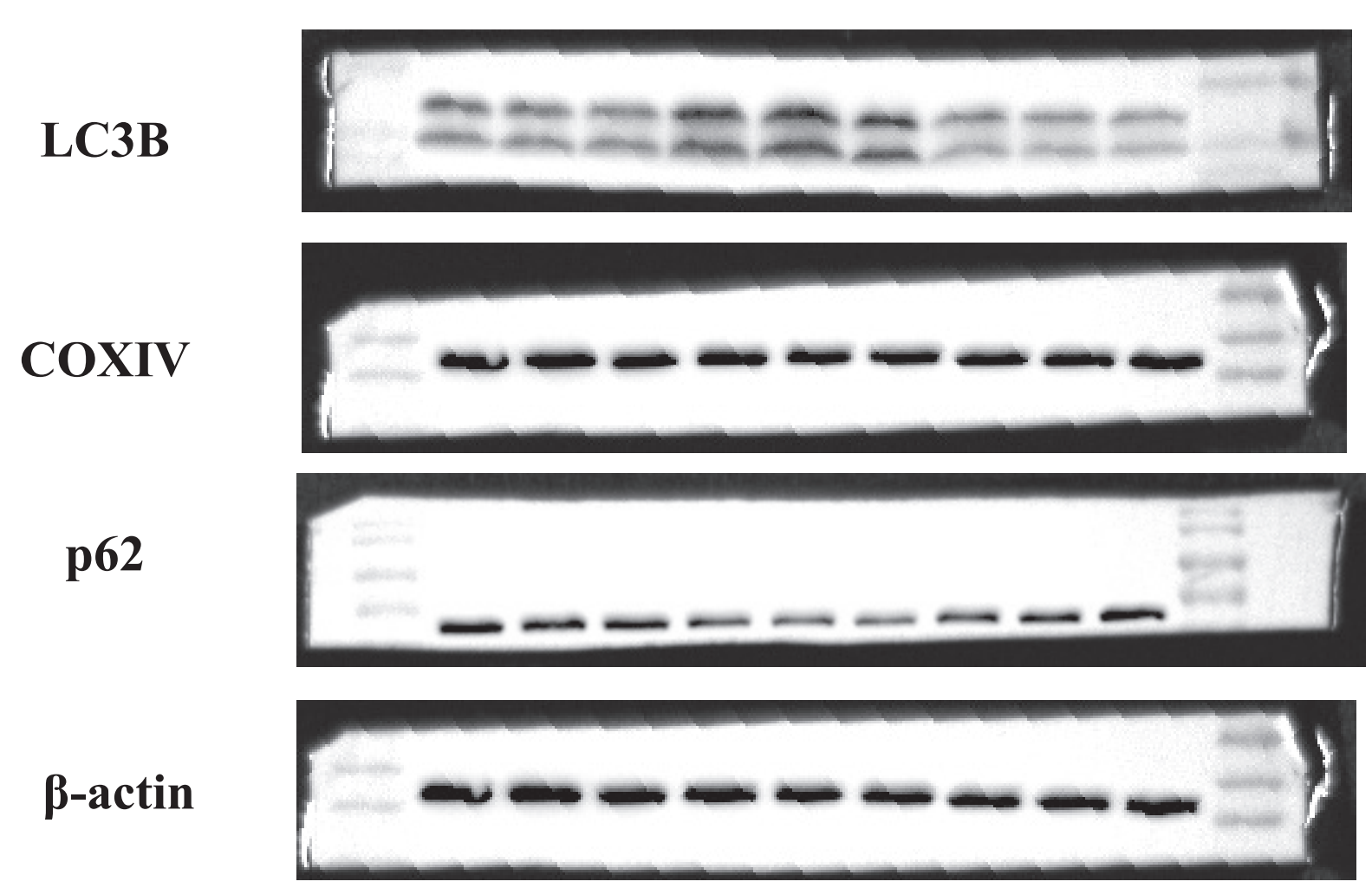

Figure 6G

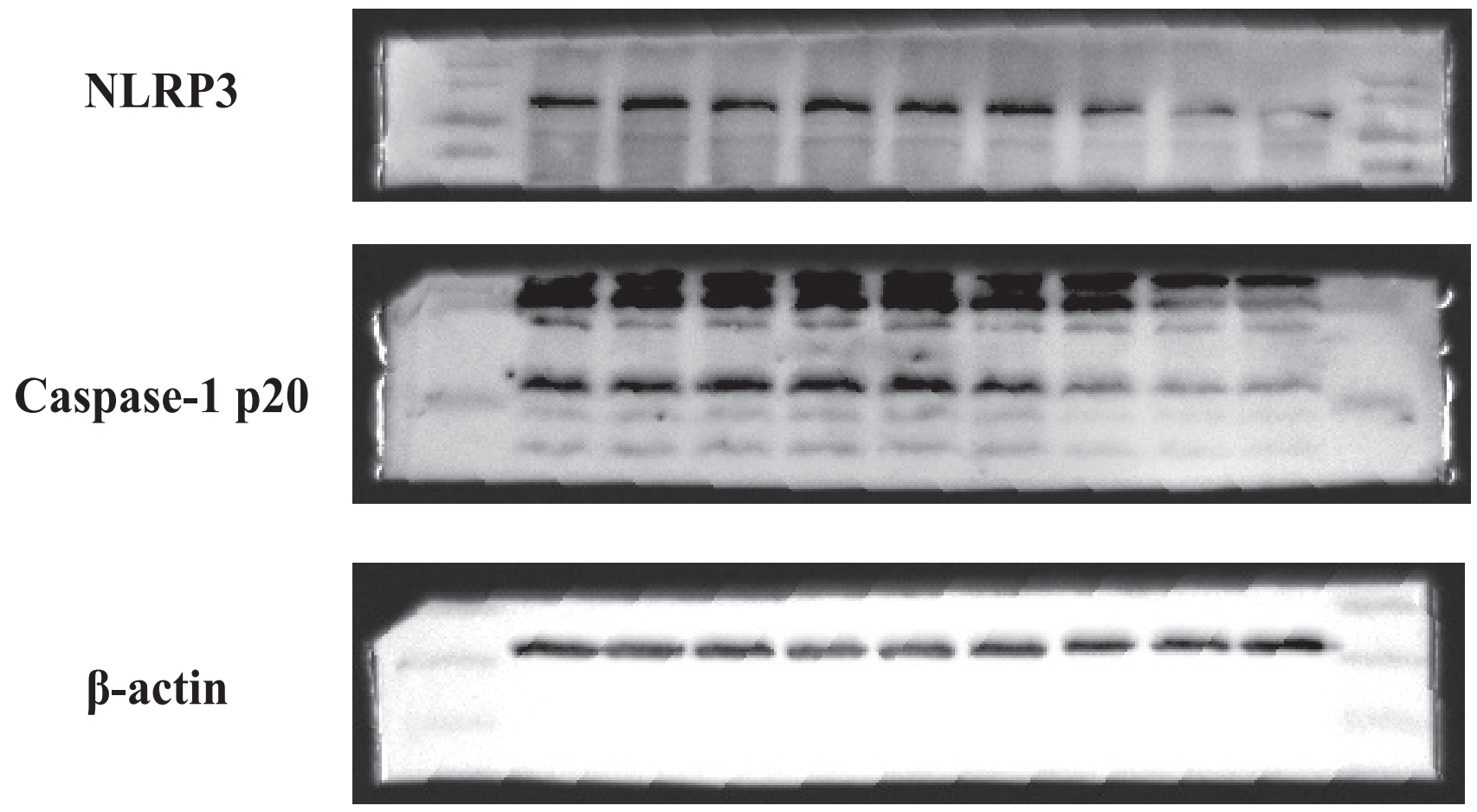

Figure S3

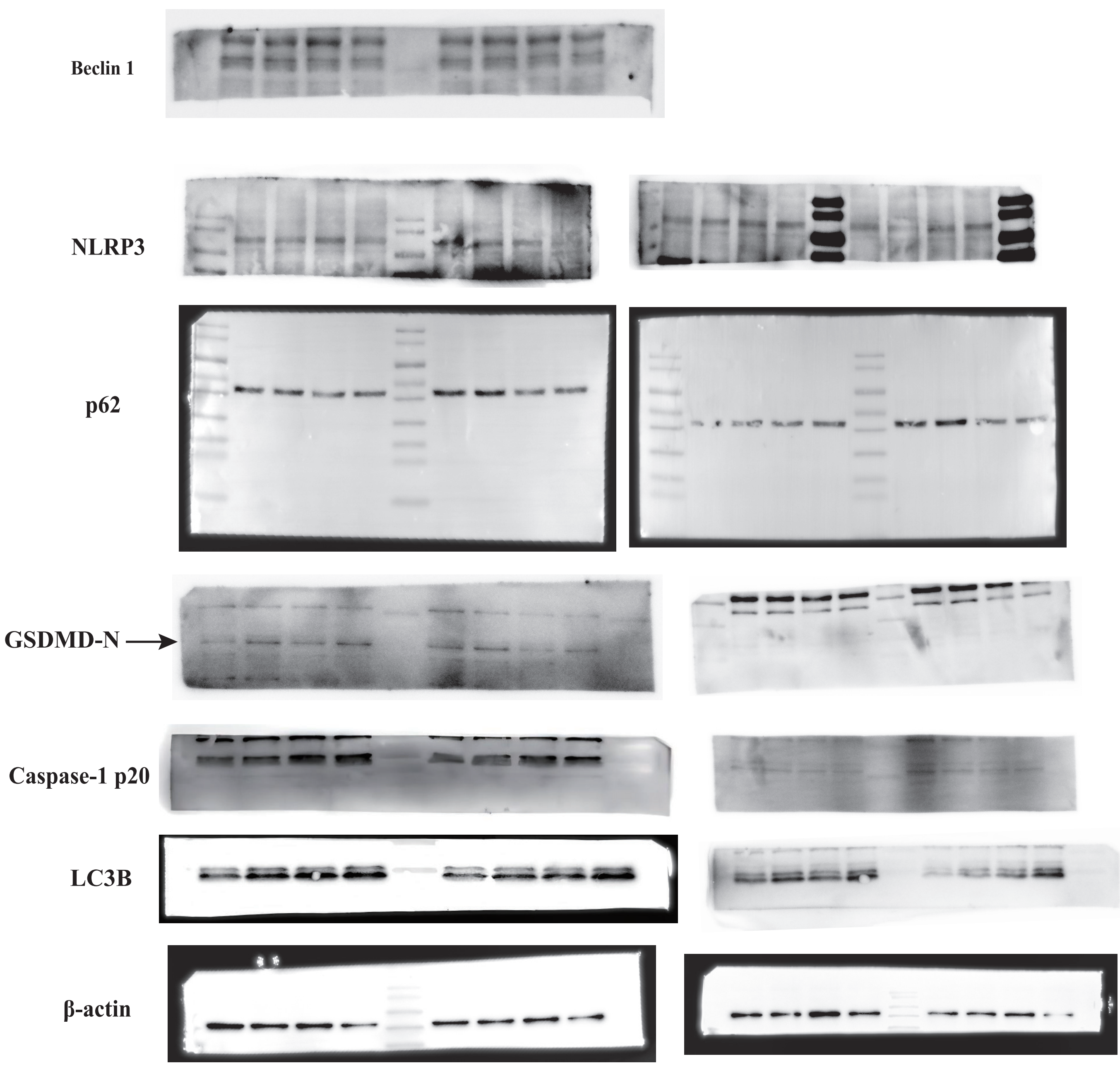

Supplement: Supplementary file 1 [file 12967_2026_8541_MOESM1_ESM.pdf]
